# No Evidence Was Found for the Presence of Terreolides, Terreumols or Saponaceolides H-S in the Fruiting Bodies of *Tricholoma terreum* (Basidiomycota, Agaricales)

**DOI:** 10.3390/molecules29081794

**Published:** 2024-04-15

**Authors:** Marco Clericuzio, Stefano Serra, Giovanni Vidari

**Affiliations:** 1 Dipartimento di Scienze ed Innovazione Tecnologica, Università del Piemonte Orientale, Via Teresa Michel 11, 15121 Alessandria, Italy; 2 Istituto di Scienze e Tecnologie Chimiche “Giulio Natta”- Consiglio Nazionale delle Ricerche (SCITEC-CNR), Via Luigi Mancinelli 7, 20131 Milano, Italy; 3 Dipartimento di Chimica, Università degli Studi di Pavia, Via Taramelli 10, 27100 Pavia, Italy; cistre@unipv.it; 4 Department of Medical Analysis, Faculty of Applied Science, Tishk International University, Erbil 44001, Iraq

**Keywords:** *Tricholoma terreum*, natural products, terpenoids, saponaceolides, bioactive metabolites, edible mushrooms

## Abstract

Two different collections of the gilled wild fungus *Tricholoma terreum*, collected in Italy, were subjected to phytochemical analysis. The fungal material was confidently identified by analysis of the ITS genomic sequences. Using both HR-LC-MS and NMR techniques, no evidence was found for the presence in the fruiting bodies of terreolides, terreumols or saponaceolides H-S, in striking contrast with the isolation of these terpenoids by Chinese authors from a mushroom collected in France and identified as *T. terreum*. The main cytotoxic terpenoid identified and isolated from the extracts of the specimens investigated in this work was the C_30_ derivative saponaceolide B, which had been previously isolated from *T. saponaceum* and other *T. terreum* collections. Although saponaceolide B is a rather labile molecule, easily degradable by heat or in acidic conditions, our study indicated that none of the extraction protocols used produced saponaceolide H-S or terreolide/terreumol derivatives, thus excluding the possibility that the latter compounds could be extraction artifacts. Considered together, these findings point to the need for the unambiguous identification of mushroom species belonging to the complex genus *Tricholoma*, characterized by high variability in the composition of metabolites. Moreover, based on our data, *T*. *terreum* must be considered an edible mushroom.

## 1. Introduction

*Tricholoma terreum* (Schaeff.: Fr.) P. Kummer (1871) is a gilled mushroom (phylum Basidiomycota, order Agaricales, family Tricholomataceae) that forms mycorrhiza with conifer trees, mainly with pines (*Pinus* sp. pl.). It was first described in Europe by Schaeffer in 1762 with the name *Agaricus terreus* and its presence was subsequently reported in other continents of the Northern Hemisphere [1]; it has also been encountered under introduced pine trees in Australia and New Zealand. The mushroom’s morphological characteristics do not vary significantly with the different places in which it grows. 

**Figure 1:**
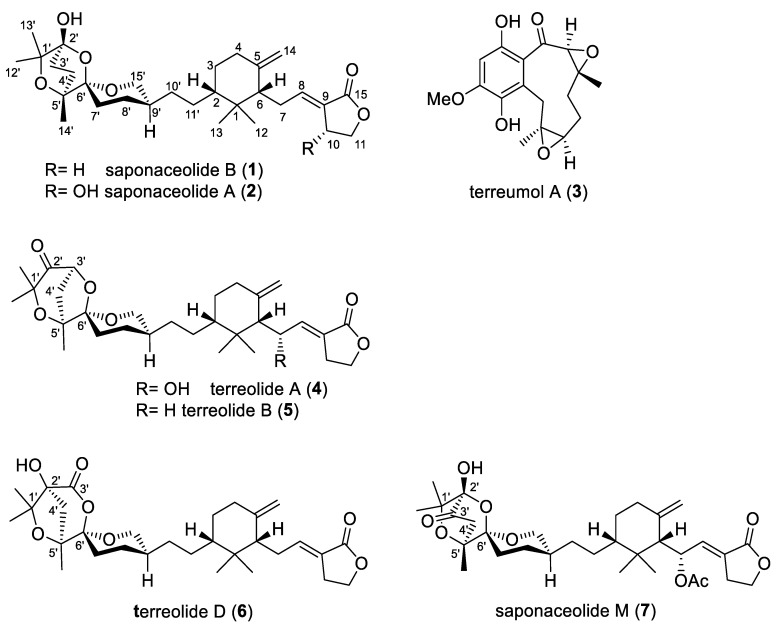
Selected terpenoids isolated from fruiting bodies of *Tricholoma terreum* and *T. saponaceum* according to published studies.

In a phytochemical investigation of *T. terreum* basidiomes reported in 1991, a dichloromethane extract was found to be strongly cytotoxic against KB and P388 cells [2]. From this extract, Geraci et al. [2] isolated the known C_30_ terpenoid saponaceolide B (**1**) (Figure 1), which was first isolated in 1991 by Vidari et al. [3] from the fruiting bodies of *T. saponaceum* (Fr.) P. Kummer (1871), together with saponaceolide A (**2**), and related saponaceolides [4,5]. 

Saponaceolide B is strongly cytotoxic and different tests on human tumor cancer cell lines have pointed to its potential development as an antitumor drug [6].

Liu et al. published a series of papers [7,8,9] on the structures of secondary metabolites isolated from an EtOAc subextract of dried fruiting bodies of a mushroom harvested in southern France, which was identified as *T. terreum*, apparently based only on morphological characteristics. In the first paper of the series, Liu et al. determined the structures of four prenylated phenols named terreumols A-D [7], which bear an unusual ten-membered monoterpenoid carbocyclic ring fused to a hydroquinone aromatic ring. One or two epoxide rings are attached to the macrocycle as in terreumol A (**3**) (Figure 1), which exhibited an antiproliferative activity comparable to that of cisplatin in a cytotoxicity assay against human cancer cells [7].

Subsequently, the same research group determined the structures of sixteen C_30_ terpenoids isolated from the same mushroom [8]. These compounds included saponaceolide B (**1**), nine new saponaceolide derivatives, H-P, and six novel related C_30_ terpenoids, named terreolides A-F, among which terreolide A (**4**) was the most abundant [8]. It has been suggested that the 5/6/7 trioxaspiroketal system of terreolides A-C, e.g., compounds **4** and **5** (Figure 1), may have resulted from a ketal reaction between C3′ and C6′ of corresponding saponaceolides, while a subsequent pinacol-like rearrangement of C4′ to C2′ may have afforded the trioxaspiroketal moiety of terreolides D-F, e.g., compound **6** (Figure 1) [8]. Finally, in the third paper of the series, three more undescribed saponaceolides, named saponaceolides Q, R and S, were isolated [9]. The Chinese authors found that the crude CHCl_3_/MeOH (1:1) extract of the fruiting bodies as well as two abundant isolated compounds, saponaceolide B (**1**) and saponaceolide M (**7**) (Figure 1), exhibited acute toxicity to mice, following oral administration, with LD_50_ values of 1.51 g kg^−1^, and 88.3 and 63.7 mg kg^−1^, respectively [8]. Moreover, compounds **1** and **7** increased serum creatine kinase levels in treated mice [8]. Based on these findings, the authors concluded that *T. terreum* should be considered toxic, causing mushroom poisoning that ultimately leads to rhabdomyolysis [8]. Indeed, this type of fungal poisoning is associated with the ingestion in consecutive meals of different mushrooms, among which *T. equestre* (L.) P. Kummer is one of the best known species [10]. 

The presumed toxicity of *T. terreum* has had a strong resonance among mycologists in Europe, where the mushroom has a long history as being edible. In fact, it is still widely consumed and has the vernacular name “moretta” [11], mainly in the alpine regions of Italy and France, where it is also sold at local markets. Moreover, there are no documented cases of poisoning due to the ingestion of *T*. *terreum* in Europe or elsewhere, and the medical literature has never reported that consumption of this mushroom induces any symptom related to rhabdomyolysis [12]. For these reasons, the presumed toxicity of *T. terreum* has been debated by different scholars [10,12,13]. 

Currently, a scientifically sound phytochemical investigation cannot be conducted without the aid of molecular data, mainly when working with Basidiomycota fruiting bodies collected in the wild. In particular, the *T. terreum* species complex has been revised with the designation of sequenced neotypes, to clarify the role of some strongly related taxa, such as *Tricholoma gausapatum* (Fr.) Quél., *T. myomyces* (Pers.) Lge, and *T. triste* (Scop.) Quél. [14].

For this reason, we decided to reinvestigate the content of secondary metabolites of *T. terreum*, using specimens firmly identified by DNA analysis and not only by morphological characteristics. Moreover, to explore the possible differences in the metabolite contents of specimens collected in different places, we examined two different batches of fruiting bodies. One was collected in a pine wood in the Bolzano province (northern Italy), which was designated as *T. terreum*_Bz, while the other was collected in a pine wood in the Grosseto province (central Italy), designated as *T. terreum*_Gr.

## 2. Results and Discussion

### 2.1. DNA Analysis

Dried specimens from the two *T. terreum* collections were sent to Molecular Solutions LLC, Portland, OR, USA (Matthew Gordon), for DNA extraction, amplification of ITS regions, and sequencing. A 695 bp sequence and a 700 bp sequence were obtained from *T. terreum*_Bz and *T. terreum*_Gr, respectively. They were submitted to the BLASTn algorithm [15] at GenBank, and both were retrieved as *Tricholoma terreum* with 100% identity relative to other sequences of the same species present in the database. The two sequences were deposited at GenBank under accession numbers PP101944 and PP101945, respectively.

### 2.2. Phytochemical Analysis

At the onset of our investigation, we verified whether the extraction procedure employed by Liu et al. [7,8,9] could alter the pattern of fungal metabolites occurring in fruiting bodies. In fact, the Chinese authors extracted air-dried fruiting bodies by means of a CHCl_3_/MeOH (1:1) mixture, although this procedure has been proven to be inappropriate for the extraction of wild mushrooms because both MeOH and HCl, usually present in CHCl_3_, often promote the formation of artifacts [16]. In addition, drying of the mushroom may also lead to important chemical changes in the metabolite contents.

**Figure 2:**
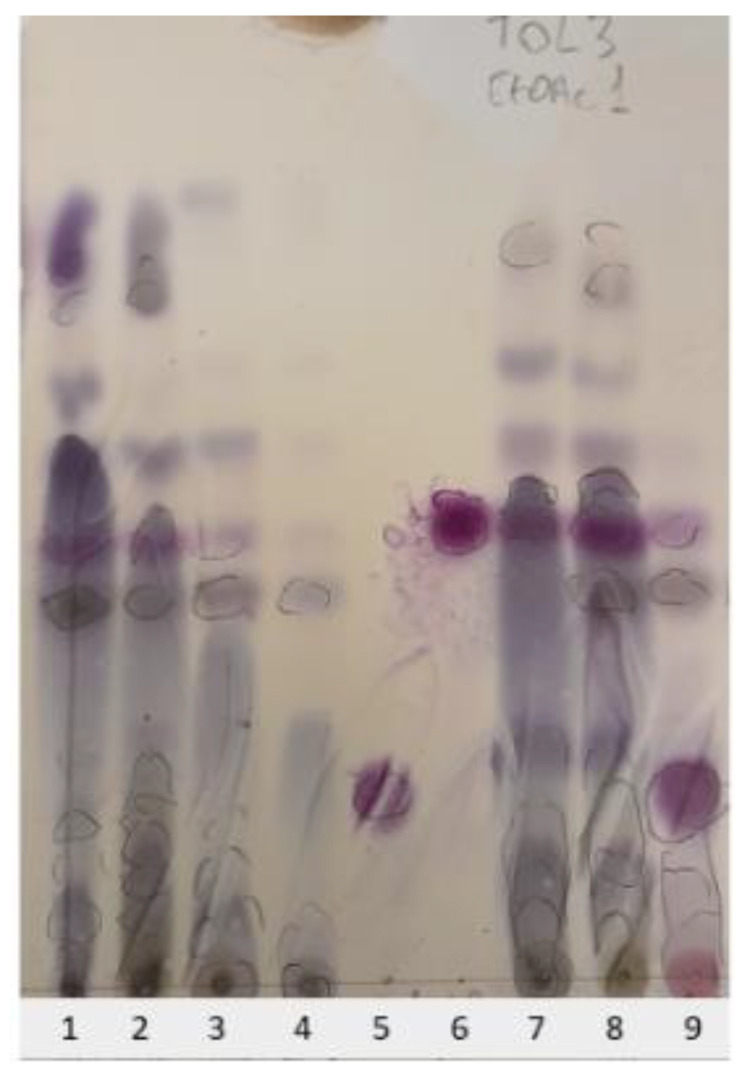
TLC analysis of the various fungal extracts, along with reference compounds, on a silica gel plate. Eluent: toluene-EtOAc, 3:1. Lane 1: air-dried specimens of *T. terreum*_Gr extracted with CHCl_3_/MeOH; lane 2: frozen specimens of *T. terreum*_Gr extracted with EtOAc; lane 3: as lane 2, extraction with 2-propanol after EtOAc; lane 4: as lane 2, extraction with MeOH after EtOAc; lane 5: saponaceolide A (**2**); lane 6: saponaceolide B (**1**); lane 7: frozen specimens of *T. terreum*_Bz extracted with EtOAc; lane 8: air-dried specimens of *T. terreum*_Bz, extracted with EtOAc; lane 9: crude EtOAc extract of *T. saponaceum*. The spots were revealed with the sulfovanillin (SV) reagent at 100 °C; the spots circled by a pencil were visible under UV light at 254 nm.

To mimic the procedure used by Liu et al. [7,8,9], the basidiomes of *T. terreum_*Gr were air-dried and extracted with CHCl_3_/MeOH (1:1). Subsequently, the crude extract was then partitioned between H_2_O and EtOAc, and the organic sub-extract was analyzed by TLC (Figure 2, lane 1). 

For comparison, a second batch of *T. terreum_*Gr basidiomes was extracted according to a procedure developed by us to avoid the formation of artifacts in mushroom extraction [16]. Thus, fresh fruiting bodies of *T. terreum_*Gr were initially frozen at −20 °C and then extracted with EtOAc (Figure 2, lane 2), followed by 2-propanol or MeOH (Figure 2, lanes 3 and 4) to also extract high polar metabolites. For *T. terreum_*Bz fruiting bodies, in one experiment they were frozen and extracted with EtOAc (extract A, Figure 2, lane 7), while in another experiment they were air-dried and then extracted with EtOAc (Figure 2, lane 8). 

The various crude extracts were compared on a TLC silica gel plate with a crude EtOAc extract of *T. saponaceum* (Figure 2, lane 9) and authentic samples of saponaceolides A (**2**) and B (**1**) (Figure 2, lanes 5 and 6). The spot patterns of the four *T. terreum* extracts in lanes 1, 2, 7, and 8 clearly indicated that the metabolite contents were almost identical, suggesting that they were not affected by the various extraction methods and the mushroom provenance. Moreover, the sulfovanillin reagent revealed the presence of saponaceolide B (**1**) in all four extracts of *T. terreum* collected in Italy as an intense purplish–reddish spot with R_f_ = 0.5, while saponaceolide A (**2**), R_f_ = 0.21, was clearly absent. The alcoholic extracts (Figure 2, lanes 3 and 4) did not contain other polar compounds in addition to those present in the EtOAc extracts, while the crude EtOAc extract of *T. saponaceum* (Figure 2, lane 9) showed, as expected, the presence of both saponaceolides A (**2**) and B (**1**). Further TLC analyses of the above-mentioned extracts, using various eluents more polar than toluene-EtOAc, 3:1, confirmed the results.

**Figure 3:**
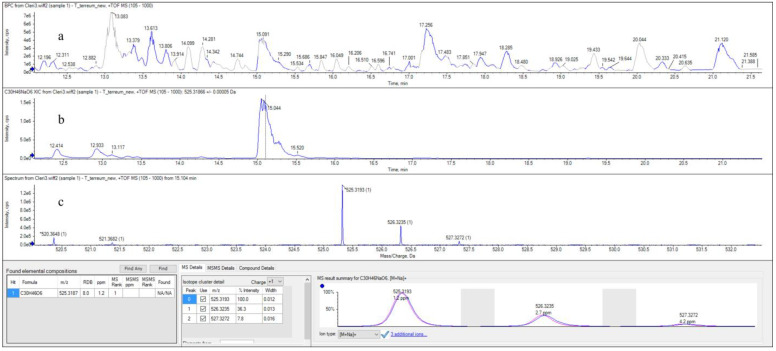
(**a**) HPLC-ESI^+^-HR QTOF MS TIC chromatogram of the crude *T. terreum*_Bz extract; (**b**) SIM extraction of the ion at *m*/*z* 525.31866, corresponding to the pseudomolecular ion [M + Na]^+^ of saponaceolide B (**1**); (**c**) cluster of the isotopic C_30_H_46_NaO_6_^+^ ions.

Crude extract A of *T. terreum*_Bz was then analyzed by HPLC-ESI^+^-HR QTOF MS to explore the presence of saponaceolides, terreolides, and terreumols. The total ion current (TIC) chromatogram of the extract is shown in Figure 3a. 

A series of single ion monitoring (SIM) experiments were performed in search of the pseudomolecular ions [M + Na]^+^ of terreolides A-F, terreumols A-D, and saponaceolides A-S. Details are reported in the Supplementary Materials (Figures S1–S7). Negative responses were obtained for all these metabolites except for saponaceolide B (**1**). The SIM search of the ion at *m*/*z* 525.31866, corresponding to the pseudomolecular ion [M + Na]^+^ (C_30_H_46_NaO_6_^+^) of saponaceolide B, allowed the extraction of a broad peak (P) from the chromatogram, at RT = 15.0–15.25 min (Figure 3b). The measured exact mass, 525.3193 a.m.u, of the monoisotopic ion ^12^C_30_^1^H_46_^23^Na^16^O_6_^+^ in the cluster of the isotopic pseudomolecular ions [M + Na]^+^ attributable to compound **1** (Figure 3c), was in agreement with the calculated accurate mass of 525.3187 a.m.u. (Δm = 1.14 ppm). 

In conclusion, these preliminary experiments indicated the absence of terreolides, terreumols, and saponaceolides, except saponaceolide B (**1**) in the extracts of *T. terreum* fruiting bodies. The striking discrepancy between our findings and those reported in a previous study prompted us to isolate the compounds expressed in *T. terreum* fruiting bodies. To this aim, fresh *T. terreum*_Bz mushrooms (1 kg) were extracted with EtOAc and the crude organic extract A (0.54% *w*/*w* of fresh mushrooms) was separated by chromatography on a silica gel column. Elution with hexane/EtOAc gave, in the first eluted fractions, a mixture of triglycerides (TG fraction) followed by free fatty acids (FA fraction) and diglycerides (DG fraction). These compounds were the main components of extract A, accounting for about 65% (*w*/*w*). 

To determine the overall composition of fatty acids in the latter fractions, a sample of TG + DG + FA fractions was treated with NaOH in MeOH/H_2_O at reflux. After acidification, the resulting mixture of free fatty acids was methylated with diazomethane, and the methyl esters, whose relative abundances are reported in Table 1, were determined by GC-MS analysis (Figures S8 and S9 in the Supplementary Materials). Oleic and linoleic acid were the main fatty acids constituents, accounting for approximately 84% of the total fatty acid content.

Our findings are consistent with a previous study conducted on the nutritional value of *T. terreum* and *T. portentosum* collected in northwest Spain [17]. It is worth noting that we have also discovered the presence of (11*Z*)-octadecenoic (asclepic, *cis*-vaccenic) acid in the significant amount of 4.3% (Table 1, entry 6). Although the presence of this uncommon fatty acid in different species of edible mushrooms has already been reported [18], its occurrence in *T. terreum* has not been described before.

After increasing the polarity of the eluent, the chromatographic separation of extract A yielded a fraction of C_28_ sterols, made almost exclusively by ergosterol (3.7% of extract A), whose chemical structure was verified by NMR and GC-MS spectra (Figures S10–S13 in the Supplementary Materials). 

Further chromatographic elution of extract A gave several minor fractions containing unidentified compounds and tentatively detected coriolic acid (**8**). They were followed by crude saponaceolide B (**1**) which was finally purified by crystallization. The ^1^H and ^13^C NMR spectra (Figures S14–S16 in the Supplementary Materials) of terpenoid **1** were superimposable with those reported in the literature [2,3], and the MS-ESI^+^ spectrum (Figure S17 in the Supplementary Materials) confirmed the expected molecular weight. In addition, the sample showed the same melting point and specific rotation as saponaceolide B isolated from *T. saponaceum* [4], proving that *T. terreum* and *T. saponaceum* produced the same enantiomer. The yield of crystallized **1** was 9.3 mg/g of extract A (0.93%, *w*/*w*), which corresponded to 47 mg/kg of *T. terreum*_Bz fresh fruiting bodies. This value was consistent with the yield of 0.7% (*w*/*w*) reported for isolated saponaceolide B by Geraci et al. [2].

**Figure 4:**
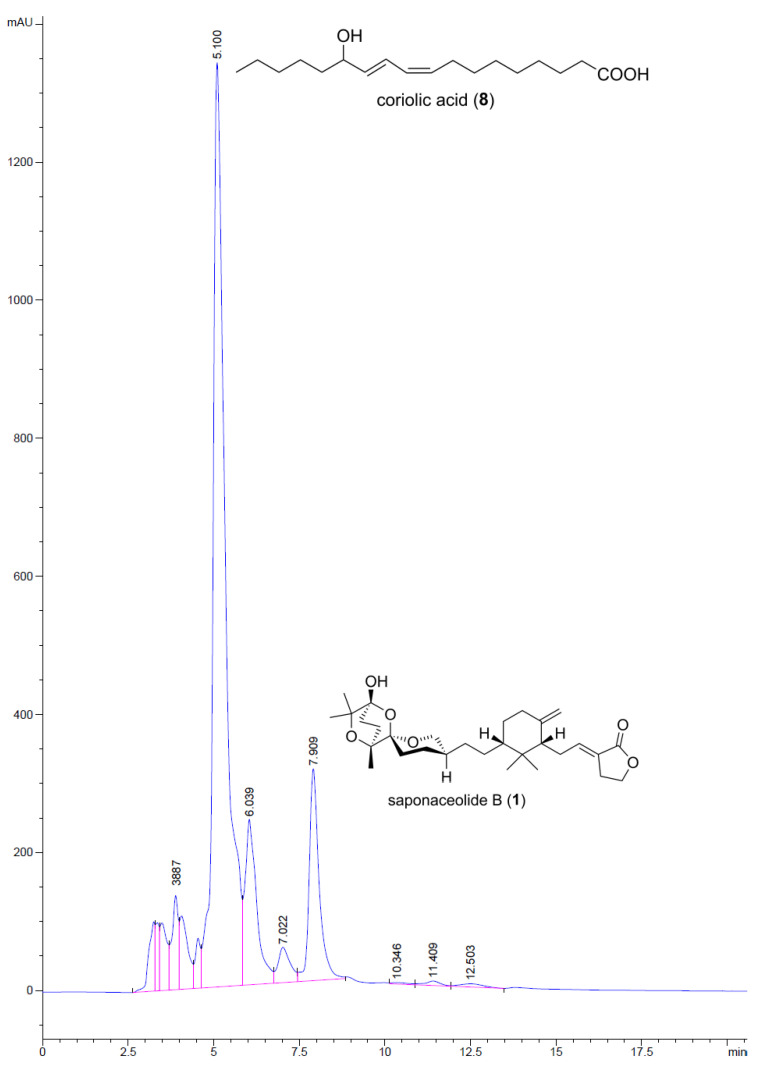
HPLC-UV chromatogram of extract A from *T. terreum*_Bz.

The HPLC-UV chromatogram of extract A from *T. terreum*_Bz is shown in Figure 4. The analysis confirmed the presence of saponaceolide B (**1**), at RT = 7.91 min, by coelution with an authentic sample. This peak was preceded by a few peaks attributable to long-chain acids and C_28_ sterols. The compound eluted as an intense peak at RT = 5.10 min was isolated from extract A by preparative TLC and identified as the bioactive (9*Z*,11*E*)-13-hydroxy-9,11-octadecadienoic acid (coriolic acid) [19] (structure **8** in Figure 4) by NMR and ESI-MS spectra (Figures S21–S23 in the Supplementary Materials).

### 2.3. Saponaceolide B Chemical Stability

**Figure 5:**
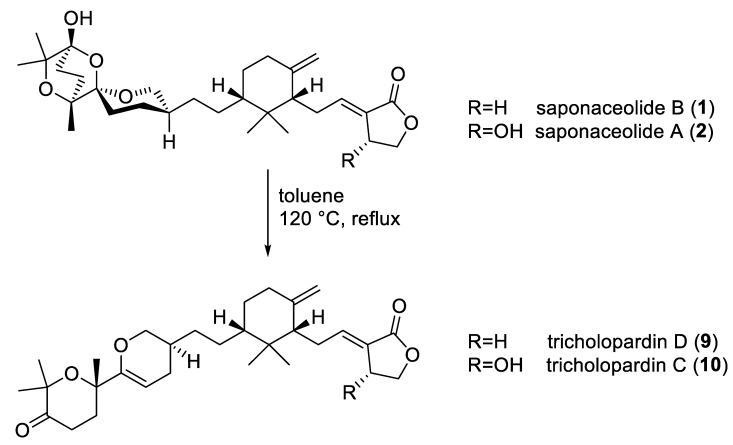
Thermal conversion of saponaceolides A and B to tricholopardins C and D.

Saponaceolide B (**1**) is highly cytotoxic [6,8]; however, it is also a rather chemically labile compound, and its structure cannot survive the traditional cooking of mushrooms in a frying pan at T > 100 °C. Therefore, any study on the thermal stability of this terpenoid may give further insights about the edibility of *T. terreum*. In this context, saponaceolides B (**1**) and A (**2**) were converted to tricholopardins D (**9**) and C (**10**), respectively, upon heating a solution in toluene at 120 °C for 12 h (Figure 5) [20]. This result indicated the thermal instability of saponaceolides A and B. 

The terpenoids **9** and **10** have already been isolated from Chinese specimens of *Tricholoma pardinum*; moreover, compound **10** exhibited significant cytotoxicity against MCF-7 cells with an IC_50_ value of 4.7 μM, by inducing apoptosis [20].

Although the saponaceolide and terreolide skeletons were suggested to derive from independent biosynthetic pathways [8], it also seemed possible that the trioxaspiroketal moiety of the terreolides could derive from that of the saponaceolides through rearrangement/oxidative reactions. However, based on the experiments previously discussed, we excluded the hypothesis that such transformations occurred in *T. terreum* basidiomes, either enzymatically or by heating. To obtain more information about the chemical stability of saponaceolide B (**1**), it was dissolved in non-stabilized CHCl_3_ and left in an open-air vial at room temperature for 1 week with the volume of the solution kept constant. Subsequent analysis of the reaction mixture by TLC, ESI-MS and NMR techniques indicated the disappearance of saponaceolide B and the formation of several products. However, neither the MS analysis nor the viewing of the ^1^H NMR signals of the mixture indicated the possible presence of terreolides, notably of terreolides B (**5**) and D (**6**) which, in principle, could have been formed by rearrangements of compound **1**. In addition, careful TLC and HPLC analysis of extract A of *T.terreum*_Bz fruiting bodies (see above) did not reveal the presence of the saponaceolide B derivatives formed in non-stabilized CHCl_3_, thus confirming that our extraction procedure did not produce artifacts. 

## 3. Materials and Methods

### 3.1. General

All solvents and reagents were of commercial quality and were purchased from Merck-Sigma-Aldrich (Milan, Italy). Reference samples of saponaceolide A (**2**) and B (**1**) were obtained by the extraction of the mushroom *T. saponaceum*, according to the procedure described by Vidari et al. [4,5].

Nuclear magnetic resonance (NMR) spectroscopy: ^1^H- and ^13^C NMR spectra and DEPT experiments: CDCl_3_ solutions at RT using a Bruker Avance AC-400 spectrometer (Billerica, MA, USA) operating at 400 and 100 MHz, respectively; ^13^C NMR spectra are proton decoupled; chemical shifts (*δ*_H_, *δ*_C_) are in ppm relative to internal SiMe_4_ (=0 ppm).

TLC: Merck silica gel *60 F*_254_ and RP18 plates (Merck Millipore, Milan, Italy); preparative TLC: Merck silica gel *60 F*_254_, thickness 2 mm (Merck Millipore, Milan, Italy).

Column chromatography: silica gel, 70–200 μm (Fluorochem, Hadfield, UK).

Melting points were measured on a Reichert apparatus (Reichert, Vienna, Austria), equipped with a Reichert microscope, and are uncorrected. 

Optical rotations were measured on a Jasco DIP-181 digital polarimeter (Jasco Europe s.r.l., Cremella (LC), Italy).

HPLC-ESI-QTOF MS spectra were recorded on a SCIEX X500B QTOF system (SCIEX, Framingham, MA 01701, USA) equipped with the Twin Sprayer ESI probe and coupled to an ExionLC^TM^ system. The chromatographic column was a Phenomenex Luna C18(2), 150 × 2.0 mm, 3 μm column (Castel Maggiore (Bo), Italy). The eluent flow was kept constant at 0.3 mL/min and the injection volume was 20 μL. The eluent gradient is reported in Table 2.

HPLC: Agilent 1260 Infinity (Agilent, Santa Clara, CA, USA) with the UV detector set at 225 nm and equipped with a YMC-CHIRAL ART Amylose-SA column (250 × 4.6 mm, 5 μm) (YMC Europe, Dinslaken, Germany). Mobile phase: hexane-isopropanol (85:15) at constant flow of 1 mL/min.

GC-MS analyses: An HP-6890 gas chromatograph, equipped with a 5973 mass detector and an HP-5MS capillary column (30 m × 0.25 mm, 0.25 μm film thickness; Hewlett Packard, Palo Alto, CA, USA), was used with the following oven temperature program: 120° (3 min)–12°/min–195° (10 min)–12°/min–300° (10 min); carrier gas: He; constant flow 1 mL/min; split ratio, 1/30.

Low resolution mass spectra were recorded on a Bruker Esquire 3000 Plus spectrometer (ESI detector) (Billerica, MA, USA) or by GC-MS analyses.

### 3.2. Fungal Material

*T. terreum*_Bz: The fruiting bodies were collected in a pine wood at Collalbo (11.458 E; 46.558 N) in the province of Bolzano, Italy on 3 August 2021 and stored at −20 °C until extraction. A lyophilized voucher specimen has been deposited at the CNR-SCITEC laboratories (via L. Mancinelli 7, Milan, Italy) with the accession number TT2022/1.

*T. terreum*_Gr: The fruiting bodies were collected in a pine wood at Castell’Azzara (11.626 E; 42.735 N) in the province of Grosseto, Italy on 23 December 2022. A dried voucher specimen has been deposited at the AGMT herbarium at Santa Croce sull’Arno, Pisa, Italy, with the accession number AGMT13648.

*T. saponaceum*: The fruiting bodies were collected at Olgiate Molgora (45°43′49.008″ N; 9°24′12.06″ E) in the province of Lecco, Italy on 6 November 2021 and stored at −20 °C until extraction with EtOAc. A lyophilized voucher specimen has been deposited at the CNR-SCITEC laboratories (via L. Mancinelli 7, Milan, Italy) with the accession number TS2021/1.

### 3.3. Extraction of Tricholoma terreum Fruiting Bodies and Isolation of Saponaceolide B (***1***)

Frozen fresh fruiting bodies of *T. terreum*_Bz (1.05 kg) were soaked in EtOAc (400 mL) and minced under the solvent. The resulting mixture was stirred for 1 h, keeping the temperature below 4 °C with the help of an external ice bath. The organic phase was removed, and further EtOAc (400 mL) was added to the mush, which was homogenized with an electric mixer. The resulting mixture was centrifuged (4 °C, 8000 rpm) and the resulting two phases were separated. The aqueous phase was extracted with EtOAc (100 mL) and the combined organic layers (extract A) were washed with brine, dried with Na_2_SO_4_ and concentrated under reduced pressure to produce a brown oil (5.4 g, residue A). A small sample (50 mg) of this oil was subjected to ESI-MS, HPLC-ESI-QTOF-MS, and HPLC-UV analyses. The remaining part of the residue (5.35 g) was chromatographed over a silica gel column at atmospheric pressure. Elution with a gradient of *n*-hexane-EtOAc-MeOH yielded, after evaporation of the fractions, 84 residues (A1–A84), whose overall weight (5.25 g) indicated the almost complete recovery of the starting sample. The residues A1–A13 (1.82 g) contained triglycerides, whereas the following residues (A14–A33) consisted of a mixture of fatty acids and diglycerides. The residues A1–A33 were combined and a sample (200 mg) of the resulting oily mixture was treated with hydroquinone (10 mg, 0.09 mmol) and a solution of NaOH (0.8 g, 20 mmol) in MeOH/H_2_O (2:1, 20 mL). The mixture was heated at reflux under nitrogen for 1 h. Subsequently, the reaction mixture was cooled to room temperature, acidified with diluted aqueous HCl and extracted with EtOAc (2 × 40 mL). The organic phase was dried with Na_2_SO_4_ and concentrated under reduced pressure. The residue was treated at 0 °C with an excess (10 mL) of a freshly prepared diazomethane solution (0.2 M in diethyl ether). The resulting mixture of fatty acid methyl esters was analyzed by GC-MS (Table 1 and Figures S8 and S9 in the Supplementary Materials). The residues A34–A40 (200 mg) contained mainly ergosterol (86% by GC-MS analysis) together with a few unidentified C_28_ sterols. This mixture was recrystallized from MeOH to give pure ergosterol which was fully characterized by NMR and GC-MS analysis (Figures S10–S13 in Supplementary Materials). The residues A65–A74 (290 mg) containing saponaceolide B (**1**) were pooled together and further chromatographed over a silica gel column. Elution with a gradient of *n*-hexane-EtOAc produced crude saponaceolide B (90 mg), which was crystallized from hexane-EtOAc (4:1) to yield 49 mg of pure terpenoid as colorless crystals, m.p. 134–136 °C (Lit.^[6]^: 134–136 °C); ${\left[\alpha \right]}_{D}^{20}$ = +16.7 (*c* 0.42, CH_2_Cl_2_), (Lit.^[6]^: ${\left[\alpha \right]}_{D}^{20}$ = +17.9 (c 1.8, CH_2_Cl_2_); ^1^H NMR (400 MHz, CDCl_3_) *δ*_H_ 6.76–6-69 (m, 1H), 4.85 (s, 1H), 4.41 (br s, 1H), 4.38 (t, *J* = 7.5 Hz, 2H), 3.70 (t, *J* = 10.9 Hz, 1H), 3.59 (dm, *J* = 10.9 Hz, 1H), 2.91–2.84 (m, 2H), 2.48–2.38 (m, 1H), 2.35–2.23 (m, 2H), 2.17 (t, *J* = 12.1 Hz, 1H ), 2.06–1.74 (m, 6H), 1.73–1.00 (m, 11H), 1.29 (s, 3H), 1.21 (s, 3H), 1.09 (s, 3H), 1.03 (s, 3H), 0.91–0.77 (m, 1H), 0.58 (s, 3H); ^13^C NMR (100 MHz, CDCl_3_) *δ*_C_ 171.3 (C), 148.1 (C), 142.2 (CH), 124.5 (C), 107.5 (CH_2_), 101.3 (C), 96.7 (C), 77.5 (C), 72.8 (C), 66.0 (CH_2_), 65.2 (CH_2_), 53.5 (CH), 48.2 (CH), 39.6 (C), 37.2 (CH_2_), 35.8 (CH), 31.7 (CH_2_), 30.1 (CH_2_), 29.3 (CH_2_), 28.6 (CH_2_), 27.9 (CH_2_), 27.7 (CH_2_), 26.8 (CH_2_), 26.5 (Me), 25.9 (Me), 25.2 (CH_2_), 24.9 (CH_2_), 22.4 (Me), 20.9 (Me), 14.8 (Me); ESI-MS: 525.4 (M + Na)^+^.

Coriolic acid (**8**) was tentatively identified in residues A41–A64 (450 mg) and A65–A74 (290 mg) along with a few unidentified fatty acid derivatives. However, a sample of coriolic acid with a purity suitable for chemical identification (Figures S21–S23 in the Supplementary Materials) was obtained by direct separation of extract A on preparative TLC plates, eluted with toluene/EtOAc, 4:1. 

A plethora of unidentified compounds was also detected in the residues A75–A84 (780 mg). However, none of the NMR and ESI-MS analyses performed on all the fractions indicated the presence of terreolides, terreumols or saponaceolides apart from saponaceolide B. 

In another experiment, a small amount (70 g) of fresh fruiting bodies of *T. terreum*_Bz was air-dried at room temperature and then extracted with EtOAc to give the corresponding extract. 

A batch (90 g) of *T. terreum*_Gr was air-dried and extracted with CHCl_3_/MeOH (1:1); subsequently, the crude extract was partitioned between H_2_O and EtOAc, and the organic phase was evaporated in a rotavapor at ≤35 °C. Another batch of the fruiting bodies was frozen at −20 °C and then extracted with EtOAc, followed by *i*-PrOH and MeOH, to give the corresponding extracts. Subsequently, all these extracts were analyzed by TLC (Figure 2).

## 4. Conclusions

The results of our study clearly indicate that neither terreolides, terreumols, nor saponaceolides H-S, isolated from a mushroom collected in southern France and identified as *Tricholoma terreum* [7,8,9], were detected in two different samples of this species collected in the wild in Italy and securely identified by ITS sequences. In striking contrast to the findings of Chinese authors [7,8,9], saponaceolide B (**1**) was the only C_30_ terpenoid present in significant and isolable amounts in the fruiting bodies, confirming the data of Geraci et al. about the contents of *T. terreum* [2]. Experimental evidence indicated that saponaceolide B (**1**) is not converted to terreolides, either enzymatically in the fruiting bodies of *T. terreum* or by heating, or under acidic conditions.

In summary, our data strongly suggest the need to base the identification of mushrooms on secure molecular data, especially as regards the species belonging to the complex genus *Tricholoma* [14]. From a chemotaxonomic perspective, compound **1** can be considered a marker of the *Tricholoma* species, having not yet been isolated from other living organisms [16]. Moreover, within the genus *Tricholoma*, the presence of saponaceolide B seems to be restricted to only a few species belonging to the sections *Contextocutis* (*T. saponaceum*) [1], *Terrea* (*T. terreum*)*,* and *Atrosquamosa* (*T. scalpturatum*) [21].

As concerns the toxicity of *T. terreum*, a value of LD_50_ = 88.3 mg/kg has been reported for the cytotoxic saponaceolide B (**1**), when administered orally in mice [8]. Considering an average weight of 60 g for a laboratory mouse, this value would approximately correspond to a toxic dose of 6.16 g for a person weighing around 70 kg. Therefore, considering that the content of saponaceolide B (**1**) in the fruiting bodies is around 47 mg/kg, serious intoxication by *T. terreum* would require the consumption by a normal adult of more than 130 kg of fresh fruiting bodies. Moreover, our study indicated the presence of other bioactive compounds in fresh fruiting bodies of *T. terreum*, namely ergosterol [22], asclepic acid [23] and coriolic acid [19,24], which are all recognized as beneficial to human health.

Overall, considering the toxicity of secondary metabolites significantly occurring in the fruiting bodies, our findings strongly indicate that *T. terreum* must be considered an edible mushroom. 

## Figures and Tables

**Table 1:** Fatty acid composition of the combined fractions of glycerides and free fatty acids extracted from *T. terreum*_Bz.

Entry	Fatty Acids (%) ^1^	Class of Fatty Acid (%) ^1^
1	C_15:0_ (0.4)	Saturated (11.4)
2	C_16:0_ (9.9)	
3	C_18:0_ (1.1)	
4	C_16:1_ (0.5)	Monounsaturated (54.1)
5	C_18:1 Δ9c_ (Oleic, 49.3)	
6	C_18:1 Δ11c_ (Asclepic, 4.3)	
7	C_18:1 Δ9c,12c_ (Linoleic, 34.5)	Polyunsaturated (34.5)

^1^ Values are expressed as percentages of total fatty acids and were determined by GC-MS analysis of the mixture of the corresponding methyl esters.

**Table 2:** Eluent gradient used to perform high-resolution HPLC -ESI^+^-QTOF MS spectra.

Time (min)	A % ^1^	B % ^2^
0	70	30
4.5	70	30
10.0	40	60
13.0	20	80
19.0	10	90
22.0	0	100
31.0	0	100

^1^ A: H_2_O with 0.1% HCOOH; ^2^ B: MeOH/MeCN, 2:1.

## Data Availability

The data supporting the findings of this study are available in the file of Supplementary Materials or from the corresponding authors.

## Supplementary Materials

The following supporting information can be downloaded at: https://www.mdpi.com/article/10.3390/molecules29081794/s1, Figure S1: SIM search for the mass peak corresponding to the formula C_30_H_44_O_7_ (terreolide A); Figure S2: SIM search for the mass peak corresponding to the formula C_17_H_20_O_6_ (terreumol A); Figure S3: SIM search for the mass peak corresponding to the formula C_32_H_46_O_9_ (saponaceolide M); Figure S4: Mass spectrum of the peak at t_R_ 13.1 min in the chromatogram of Figure 3; Figure S5: Mass spectrum of the peak at t_R_ 13.6 min in the chromatogram of Figure 3; Figure S6: Mass spectrum of the peak at t_R_ 17.3 min in the chromatogram of Figure 3; Figure S7: Mass spectrum of the peak at t_R_ 21.1 min in the chromatogram of Figure 3; Figure S8: GC-MS analysis of the fatty acid content of the *T. terreum*_Bz extract; Figure S9: GC-MS analysis of the fatty acid content of the *T. terreum*_Bz extract after addition of a reference standard of asclepic acid; Figure S10: ^1^H NMR spectrum of ergosterol extracted from *T. terreum* _Bz; Figure S11: ^13^C NMR spectrum of ergosterol extracted from *T. terreum*_Bz; Figure S12: ^13^C DEPT135 NMR spectrum of ergosterol extracted from *T. terreum*_Bz; Figure S13: GC-MS spectrum of ergosterol extracted from *T. terreum*_Bz; Figure S14: ^1^H NMR spectrum of saponaceolide B; Figure S15: ^13^C NMR spectrum of saponaceolide B; Figure S16: NMR-DEPT experiment of saponaceolide B; Figure S17: ESI-MS analysis of saponaceolide B; Figure S18: HPLC analysis of a reference standard of saponaceolide A; Figure S19: HPLC analysis of a reference standard of saponaceolide B; Figure S20: HPLC analysis *T. terreum* _Bz extract; Figure S21: ^1^H NMR spectrum of the coriolic acid extracted from *T. terreum*_Bz; Figure S22: ^13^C NMR spectrum of the coriolic acid extracted from *T. terreum*_Bz ; Figure S23: ESI-MS spectrum of the coriolic acid extracted from *T. terreum*_Bz.

## References

[1] Christensen M., Heilmann-Clausen J. (2013). Fungi of Northern Europe.

[2] Geraci C., Piattelli M., Tringali C. (1991). Applications of two-dimensional NMR in spectral assignments of the cytotoxic triterpene saponaceolide B. Magn. Reson. Chem..

[3] De Bernardi M., Garlaschelli L., Toma L., Vidari G., Vita-Finzi P. (1991). Fungal metabolites XXVI: The structure of saponaceolides B, C and D, new C-30 terpenoids from *Tricholoma saponaceum*. Tetrahedron.

[4] De Bernardi M., Garlaschelli L., Gattl G., Vidari G., Finzi P.V. (1988). Fungal metabolites XXII: The unprecedented structure of saponaceolide a, a cytotoxic C-30 terpenoid from *Tricholoma saponaceum*. Tetrahedron.

[5] Gozzini D., Mellerio G.G., Gilardoni G., Clericuzio M., Vidari G. (2018). New terpenoids from *Tricholoma saponaceum*. Nat. Prod. Commun..

[6] Vidari G., Lanfranchi G., Sartori P., Serra S. (1995). Saponaceolides: Differential cytotoxicity and enantioselective synthesis of the right-hand lactone moiety. Tetrahedron-Asymmetry.

[7] Yin X., Feng T., Li Z.-H., Dong Z.-J., Li Y., Liu J.K. (2013). Highly oxygenated meroterpenoids from fruiting bodies of the mushroom *Tricholoma terreum*. J. Nat. Prod..

[8] Yin X., Feng T., Shang J.-H., Zhao Y.-L., Wang F., Li Z.-H., Dong Z.-J., Luo X.-D., Liu J.-K. (2014). Chemical and toxicological investigations of a previously unknown poisonous european mushroom *Tricholoma terreum*. Chem. Eur. J..

[9] Feng T., He J., Ai H.-L., Huang R., Li Z.-H., Liu J.-K. (2015). Three new triterpenoids from european mushroom *Tricholoma terreum*. Nat. Prod. Bioprospect..

[10] Saviuc P., Danel V. (2006). New syndromes in mushroom poisoning. Toxicol. Rev..

[11] Boa E. (2004). Wild Edible Fungi: A Global Overview of Their Use and Importance to People.

[12] Davoli P., Floriani M., Assisi F., Kob K., Sitta N. (2016). Comment on “chemical and toxicological investigations of a previously unknown poisonous european mushroom *Tricholoma terreum*”. Chem. Eur. J..

[13] Yin X., Feng T., Li Z.-H., Liu J.-K. (2016). Response to the “comment on chemical and toxicological investigations of a previously unknown poisonous european mushroom *Tricholoma terreum*”. Chem. Eur. J..

[14] Heilmann-Clausen J., Christensen M., Frøslev T.G., Kjøller R. (2017). Taxonomy of *Tricholoma* in northern europe based on its sequence data and morphological characters. Persoonia—Mol. Phylogeny Evol. Fungi.

[15] Altschul S.F., Gish W., Miller W., Myers E.W., Lipman D.J. (1990). Basic local alignment search tool. J. Mol. Biol..

[16] Clericuzio M., Mellerio G.G., Finzi P.V., Vidari G. (2018). Secondary metabolites isolated from *Tricholoma* species (basidiomycota, tricholomatacee): A review. Nat. Prod. Commun..

[17] Díez V.A., Alvarez A. (2001). Compositional and nutritional studies on two wild edible mushrooms from northwest spain. Food Chem..

[18] Pedneault K., Angers P., Gosselin A., Tweddell R.J. (2008). Fatty acid profiles of polar and neutral lipids of ten species of higher basidiomycetes indigenous to eastern canada. Mycol. Res..

[19] Ko Y.-C., Choi H.S., Kim J.-H., Kim S.-L., Yun B.-S., Lee D.-S. (2020). Coriolic acid (13-(*S*)-hydroxy-9*Z*,11*E*-octadecadienoic acid) from glasswort (*Salicornia herbacea* L.) suppresses breast cancer stem cell through the regulation of c-Myc. Molecules.

[20] Shi C., Peng Y.-L., He J., Li Z.-H., Liu J.-K., Feng T. (2021). Structures, chemical conversions, and cytotoxicity of tricholopardins C and D, two *Tricholoma* triterpenoids from the wild mushroom *Tricholoma pardinum*. Nat. Prod. Bioprospect..

[21] Pang Z., Bergquist K.-E., Sterner O. (1994). The isolation of a new isochromanone from injured fruit bodies of *Tricholoma scalpturatum*. Acta Chem. Scand..

[22] Rangsinth P., Sharika R., Pattarachotanant N., Duangjan C., Wongwan C., Sillapachaiyaporn C., Nilkhet S., Wongsirojkul N., Prasansuklab A., Tencomnao T. (2023). Potential beneficial effects and pharmacological properties of ergosterol, a common bioactive compound in edible mushrooms. Foods.

[23] Field C.J., Blewett H.H., Proctor S., Vine D. (2009). Human health benefits of vaccenic acid. Appl. Physiol. Nutr. Metab..

[24] Gargouri M., Legoy M.D. (1997). Chemoenzymatic production of (+)-coriolic acid from trilinolein: Coupled synthesis and extraction. J. Am. Oil Chem. Soc..

