# Supplementary material for: No Evidence Was Found for the Presence of Terreolides, Terreumols or Saponaceolides H-S in the Fruiting Bodies of Tricholoma terreum (Basidiomycota, Agaricales)
Source: Molecules. 2024 Apr 15;29(8):1794. doi: 10.3390/molecules29081794 (PMC11052463; doi:10.3390/molecules29081794)
Supplement: Supplementary file 1 [file molecules-29-01794-s001.zip › molecules-2938294-supplementary.pdf]

Supplementary Materials

No Evidence Was Found for the Presence of Terreolides, Terreumols or Saponaceolides H-S in the Fruiting Bodies of *Tricholoma terreum* (Basidiomycota, Agaricales)

Table of contents:

1. Figure S1: SIM search for the mass peak corresponding to the formula $C_{30}H_{44}O_7$ (terreolide A)
2. Figure S2: SIM search for the mass peak corresponding to the formula $C_{17}H_{20}O_6$ (terreumol A)
3. Figure S3: SIM search for the mass peak corresponding to the formula $C_{32}H_{46}O_9$ (saponaceolide M)
4. Figure S4: Mass spectrum of the peak at t_R 13.1 minutes in the chromatogram of fig. 3. Best fit is found for a molecular formula $C_{18}H_{30}O_2$
5. Figure S5: Mass spectrum of the peak at t_R 13.6 minutes in the chromatogram of fig. 3. Best fit is found for a molecular formula $C_{18}H_{30}O_3$
6. Figure S6: Mass spectrum of the peak at t_R 17.3 minutes in the chromatogram of fig. 3. Best fit is found for a molecular formula $C_{28}H_{42}O$.
7. Figure S7: Mass spectrum of the peak at t_R 21.1 minutes in the chromatogram of fig. 3. Best fit is found for a molecular formula $C_{28}H_{42}O$
8. Figure S8: GC-MS analysis of the fatty acid content of the *T. terreum*_Bz extract
9. Figure S9: GC-MS analysis of the fatty acid content of the *T. terreum*_Bz extract after addition of a reference standard of asclepic acid.
10. Figure S10: ^1H NMR spectrum of ergosterol extracted from *T. terreum* _Bz
11. Figure S11: ^{13}C NMR spectrum of ergosterol extracted from *T. terreum* _Bz
12. Figure S12: ^{13}C DEPT135 NMR spectrum of ergosterol extracted from *T. terreum* _Bz
13. Figure S13: GC-MS spectrum of ergosterol extracted from *T. terreum* _Bz
14. Figure S14: ^1H NMR spectrum of saponaceolide B
15. Figure S15: ^{13}C NMR spectrum of saponaceolide B
16. Figure S16: NMR-DEPT experiment of saponaceolide B
17. Figure S17: ESI-MS analysis of saponaceolide B
18. Figure S18: HPLC analysis of a reference standard of saponaceolide A
19. Figure S19: HPLC analysis of a reference standard of saponaceolide B
20. Figure S20: HPLC analysis *T. terreum* _Bz extract

21. Figure S21: ^{1}H NMR spectrum of the coriolic acid extracted from *T. terreum* _Bz
22. Figure S22: ^{13}C NMR spectrum of the coriolic acid extracted from *T. terreum* _Bz
23. Figure S23: ESI-MS spectrum of the coriolic acid extracted from *T. terreum* _Bz

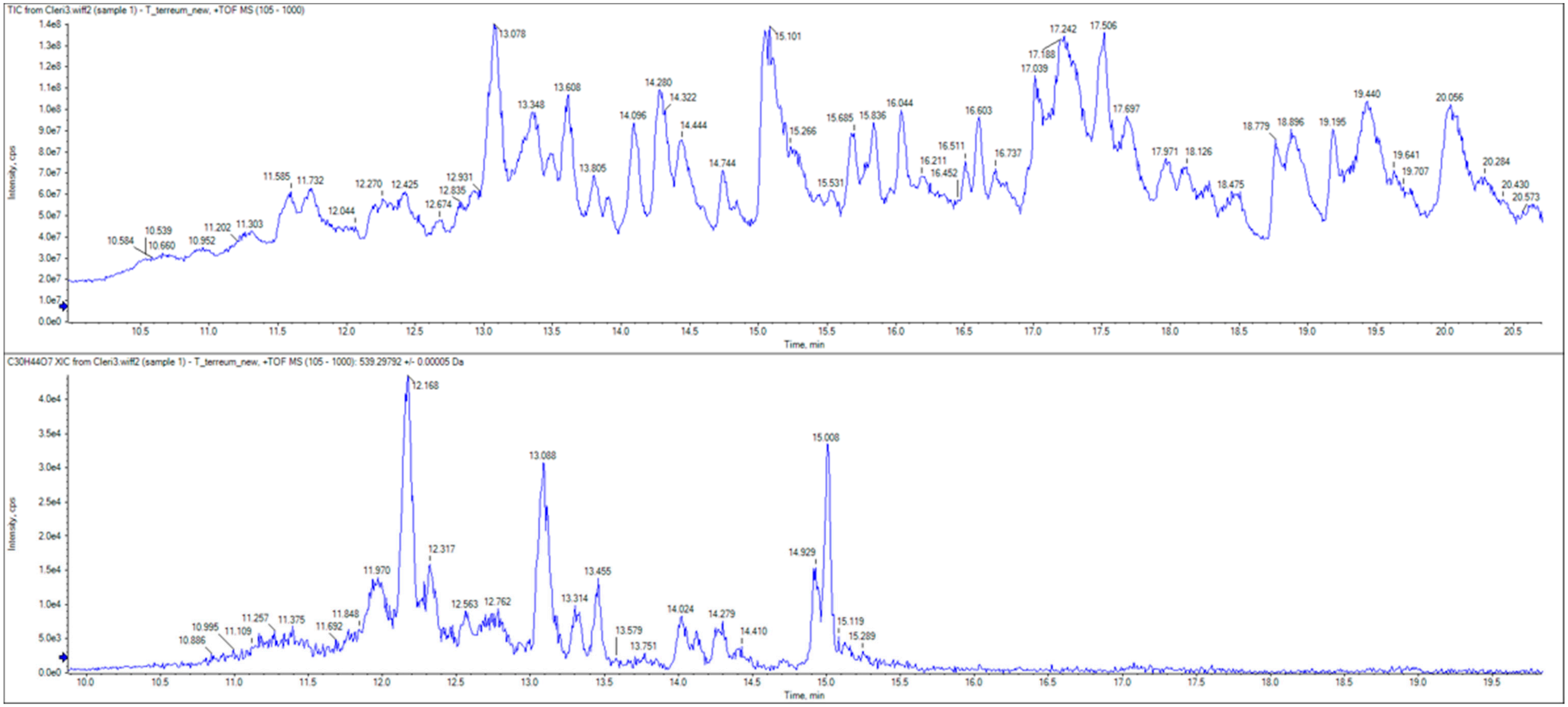

Figure S1: SIM search for the mass peak corresponding to the formula $C_{30}H_{44}O_7$ (terreolide A), in the HR-ESI$^+$ chromatogram of *T. terreum*_Bz. Note that the intensity of the retrieved peaks is almost four orders of magnitude lower than the peaks in the TIC chromatogram. As a consequence, we consider this as a negative result.

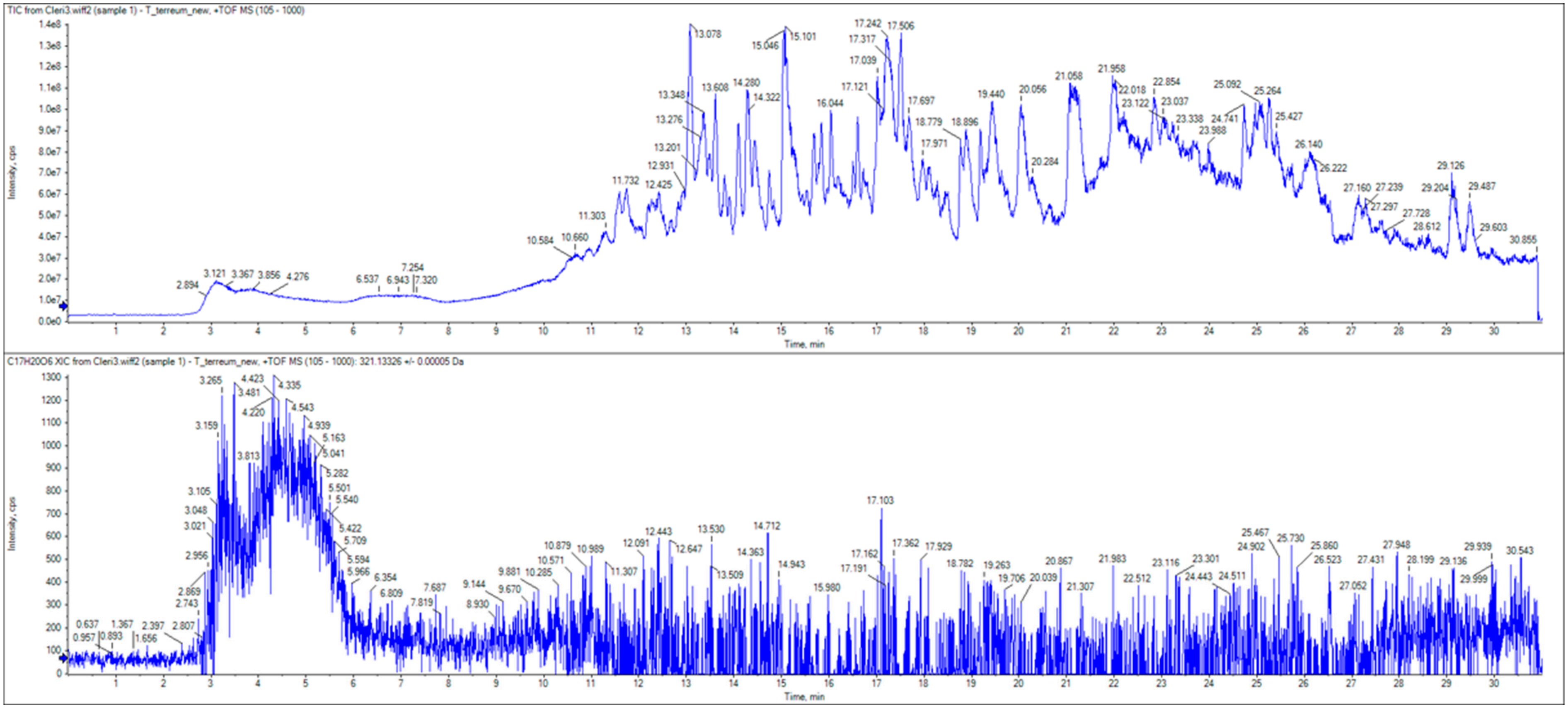

Figure S2: SIM search for the mass peak corresponding to the formula $C_{17}H_{20}O_6$ (terreumol A).

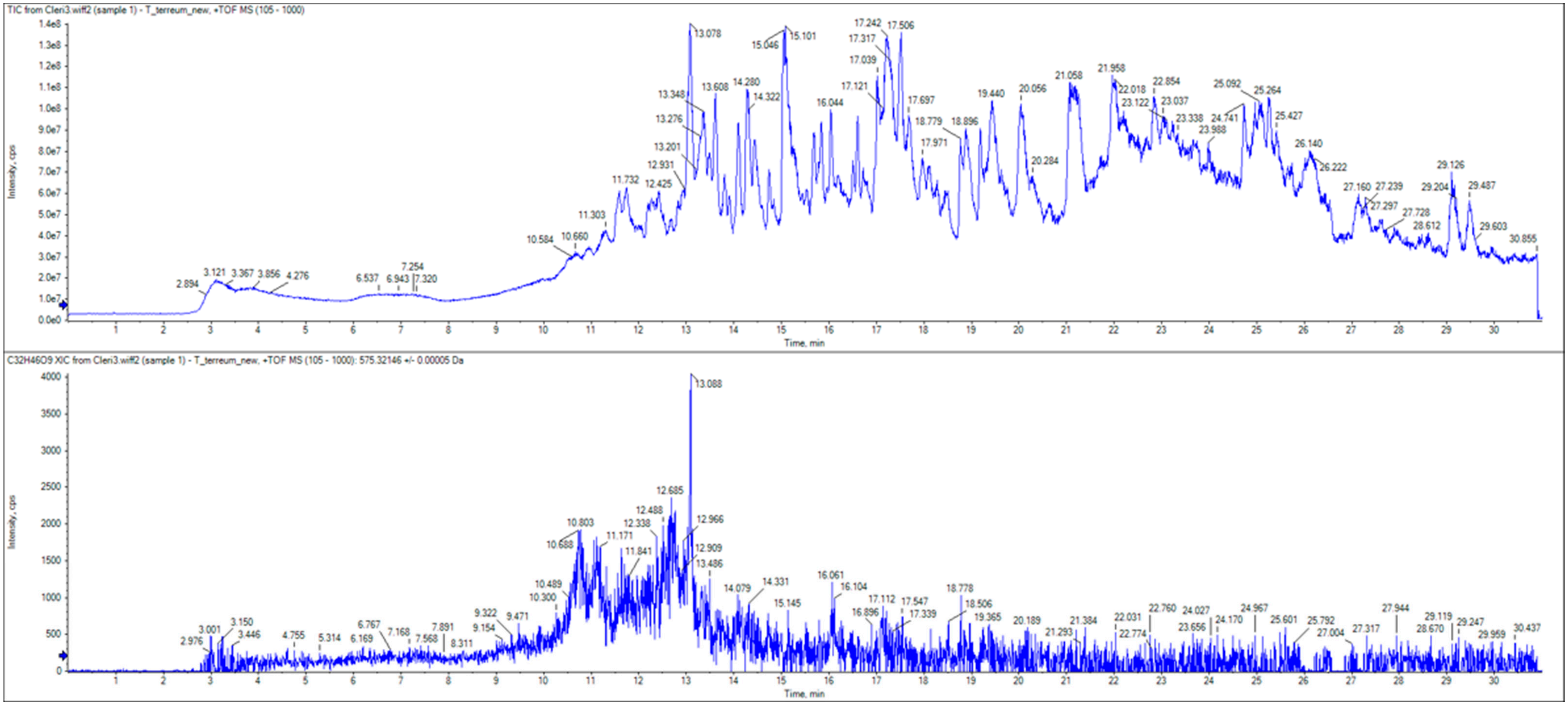

Figure S3: SIM search for the mass peak corresponding to the formula $C_{32}H_{46}O_9$ (saponaceolide M).

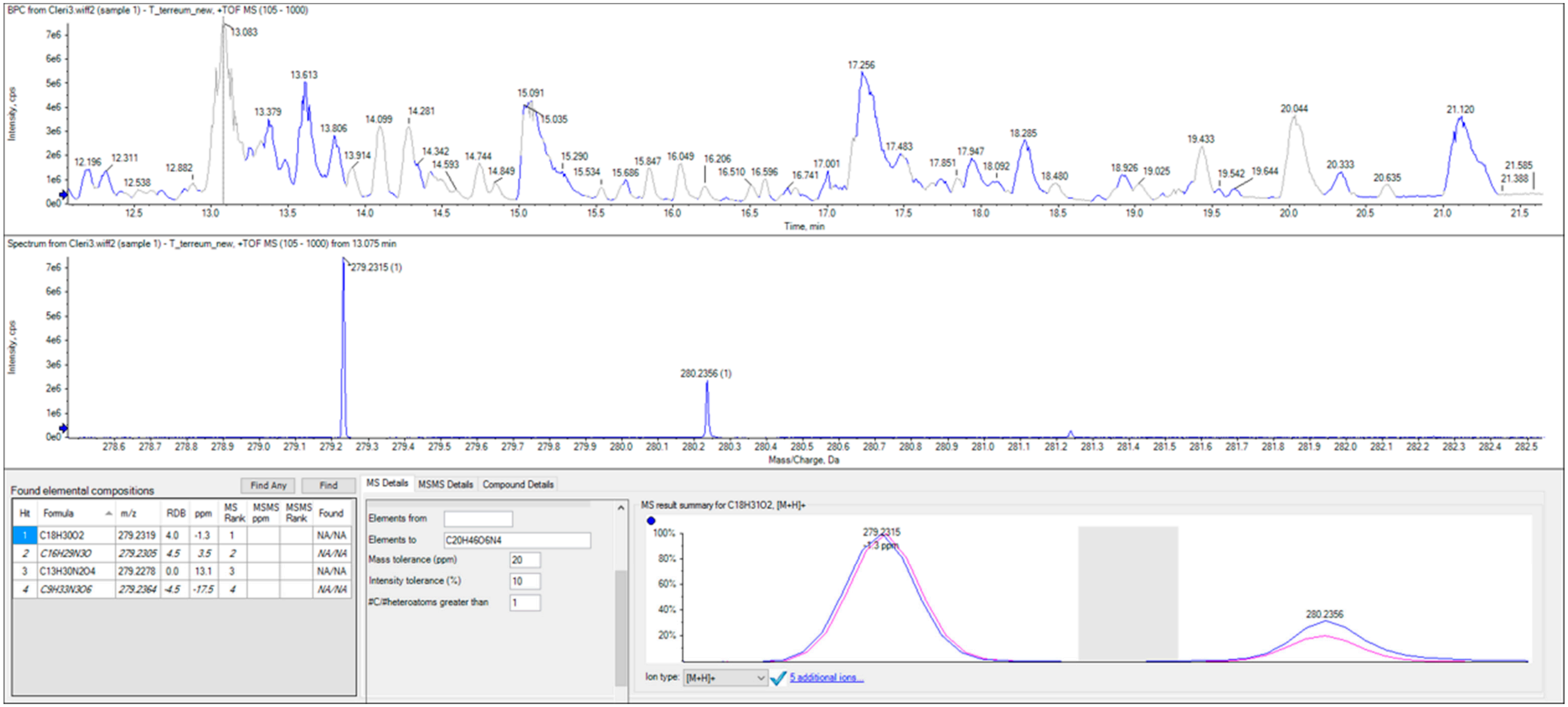

Hit	Formula	m/z	RDB	ppm	MS Rank	MSMS ppm	MSMS Rank	Found
1	C18H30O2	279.2319	4.0	-1.3	1			NA/NA
2	C16H29N3O	279.2305	4.5	3.5	2			NA/NA
3	C13H30N2O4	279.2278	0.0	13.1	3			NA/NA
4	C9H33N3O6	279.2364	-4.5	-17.5	4			NA/NA

Figure S4: Mass spectrum of the peak at t_R 13.1 minutes in the chromatogram of fig. 3. Best fit is found for a molecular formula $C_{18}H_{30}O_2$.

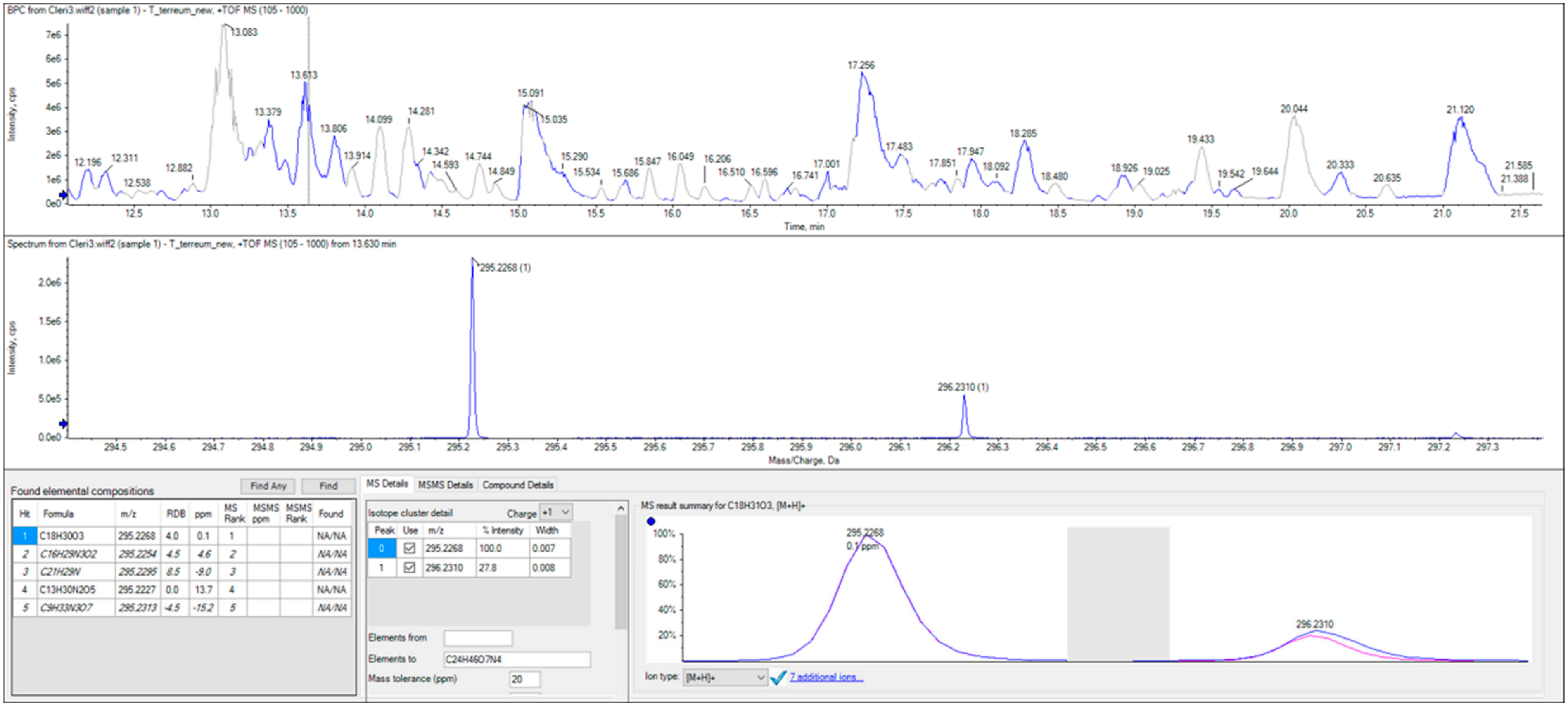

Figure S5: Mass spectrum of the peak at t_R 13.6 minutes in the chromatogram of fig. 3. Best fit is found for a molecular formula $C_{18}H_{30}O_3$.

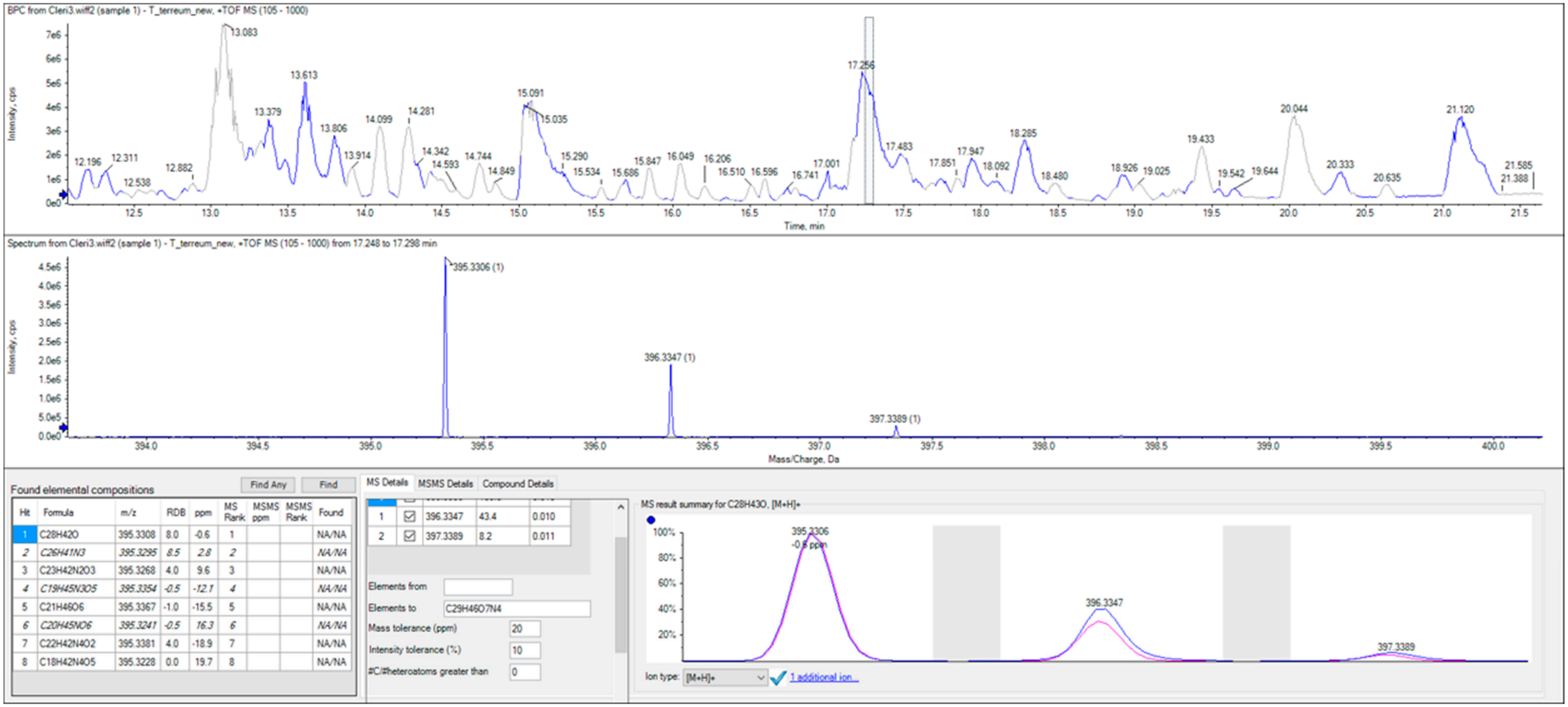

Figure S6: Mass spectrum of the peak at t_R 17.3 minutes in the chromatogram of fig. 3. Best fit is found for a molecular formula $C_{28}H_{42}O$.

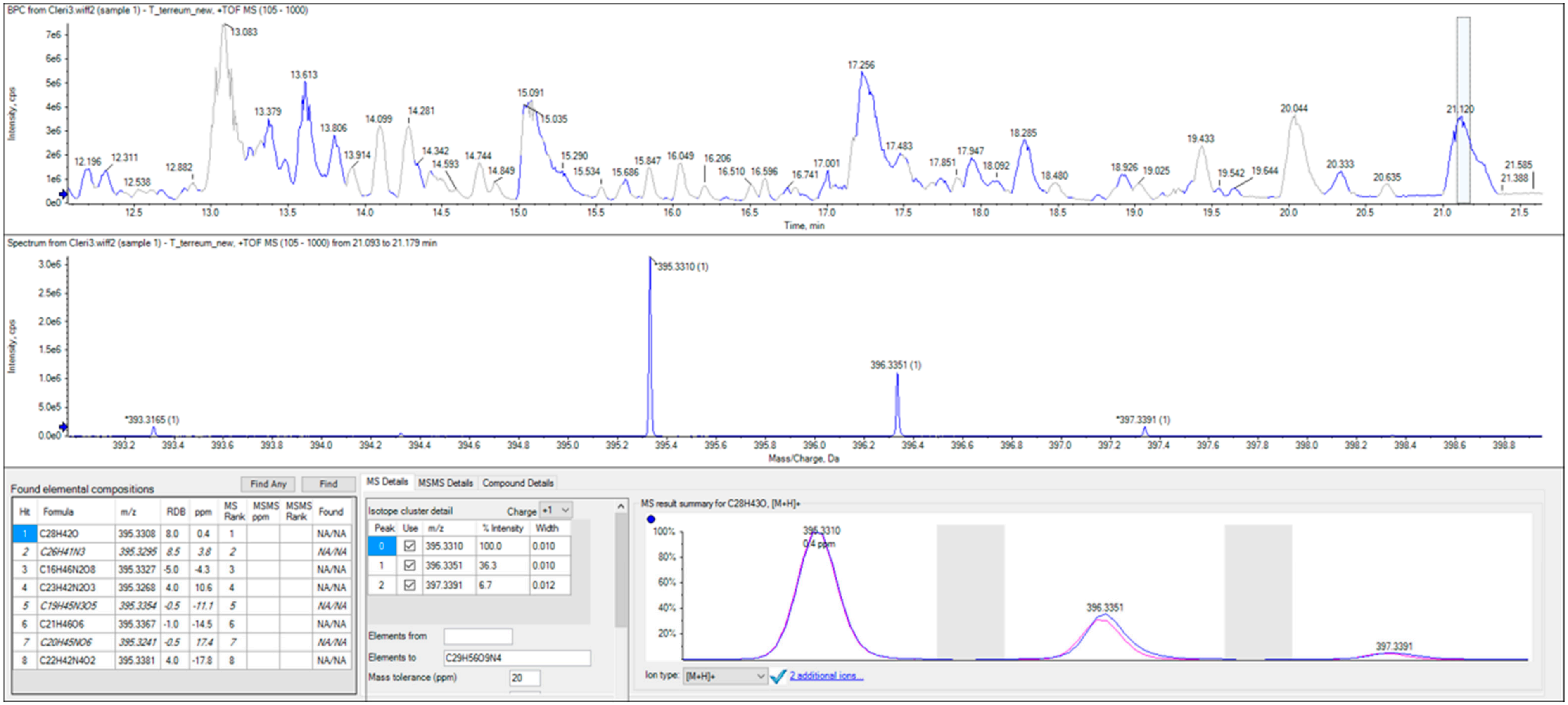

Figure S7: Mass spectrum of the peak at t_R 21.1 minutes in the chromatogram of fig. 3. Best fit is found for a molecular formula $C_{28}H_{42}O$

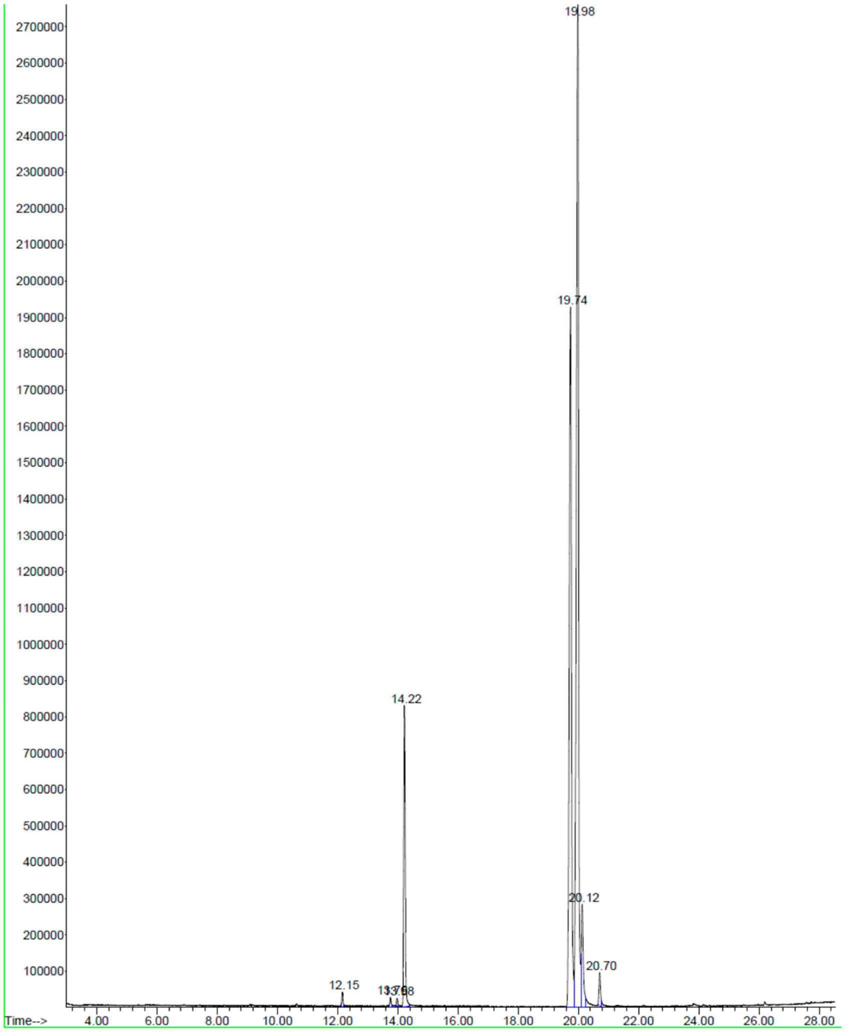

peak #	R.T. min	first scan	max scan	last scan	PK TY	peak height	corr. area	corr. % max.	% of total
1	12.153	1709	1715	1727	M2	40583	1046539	0.74%	0.366%
2	13.755	2006	2015	2024	M6	26051	816460	0.58%	0.285%
3	13.974	2048	2056	2064	M5	21426	627305	0.44%	0.219%
4	14.220	2087	2102	2133	M2	830081	28337847	20.10%	9.901%
5	19.742	3015	3035	3052	M2	1935090	98870253	70.12%	34.544%
6	19.980	3052	3071	3086	M	2766602	140998092	100.00%	49.263%
7	20.126	3087	3093	3109	M3	282116	12236644	8.68%	4.275%
8	20.702	3172	3180	3189	M4	91188	3283962	2.33%	1.147%

t_R 12.15 min: pentadecanoic acid methyl ester
t_R 13.75 min and 13.97 min: palmitoleic acid methyl ester isomers
t_R 14.22 min: palmitic acid methyl ester
t_R 19.74 min: linoleic acid methyl ester
t_R 19.98 min: oleic acid methyl ester
t_R 20.13 min: asclepic acid methyl ester
t_R 20.70 min: stearic acid methyl ester

Figure S8: GC-MS analysis of the fatty acid content of the *T. terreum*_Bz extract.

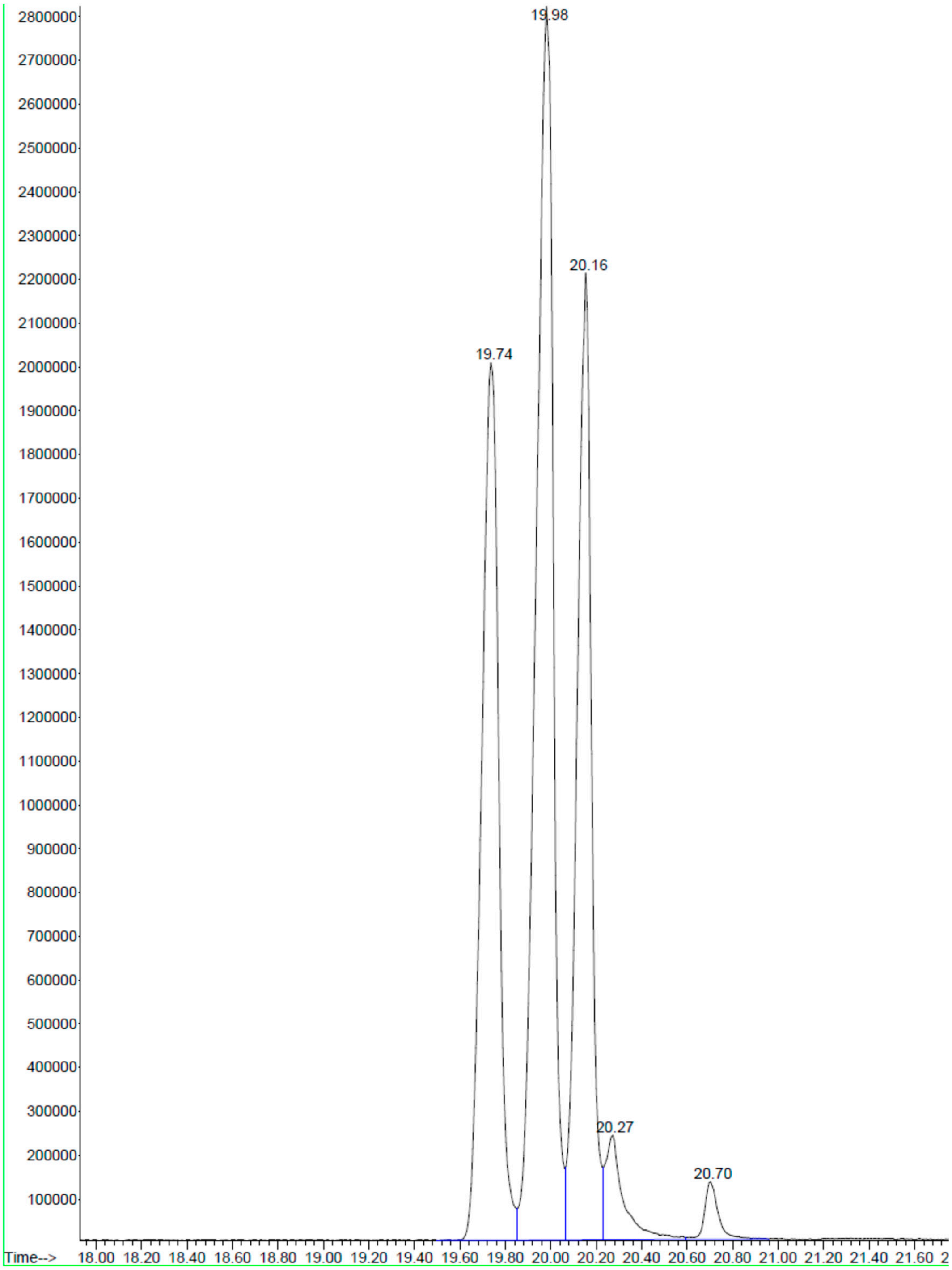

Figure S9: GC-MS analysis of the fatty acid content of the *T. terreum*_Bz extract after addition of a reference standard of asclepic acid (t_R 20.16 min) containing the corresponding *trans* isomer (vaccenic acid, t_R 20.27 min).

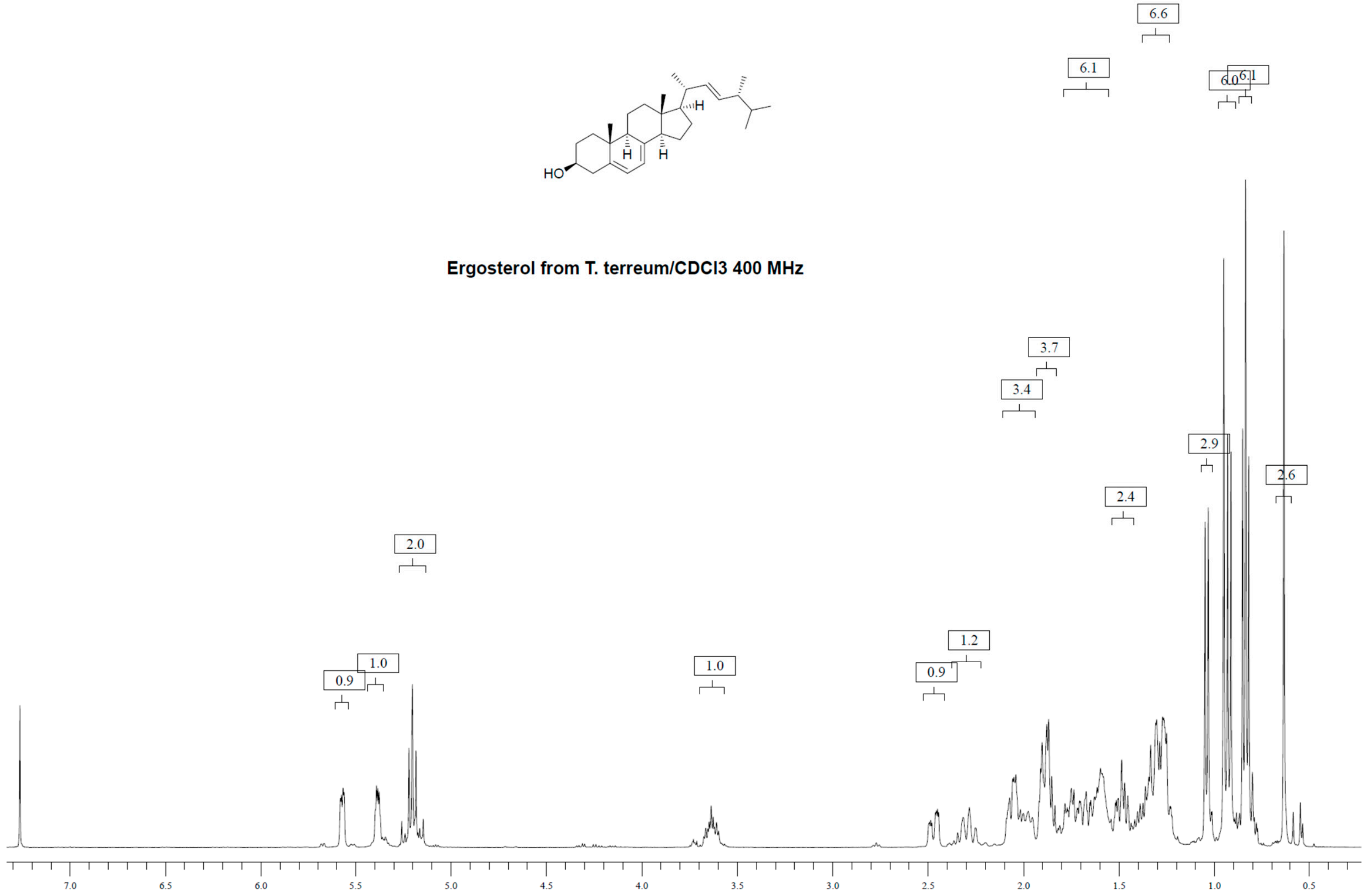

Figure S10: ^{1}H NMR spectrum of ergosterol extracted from *T. terreum* _Bz.

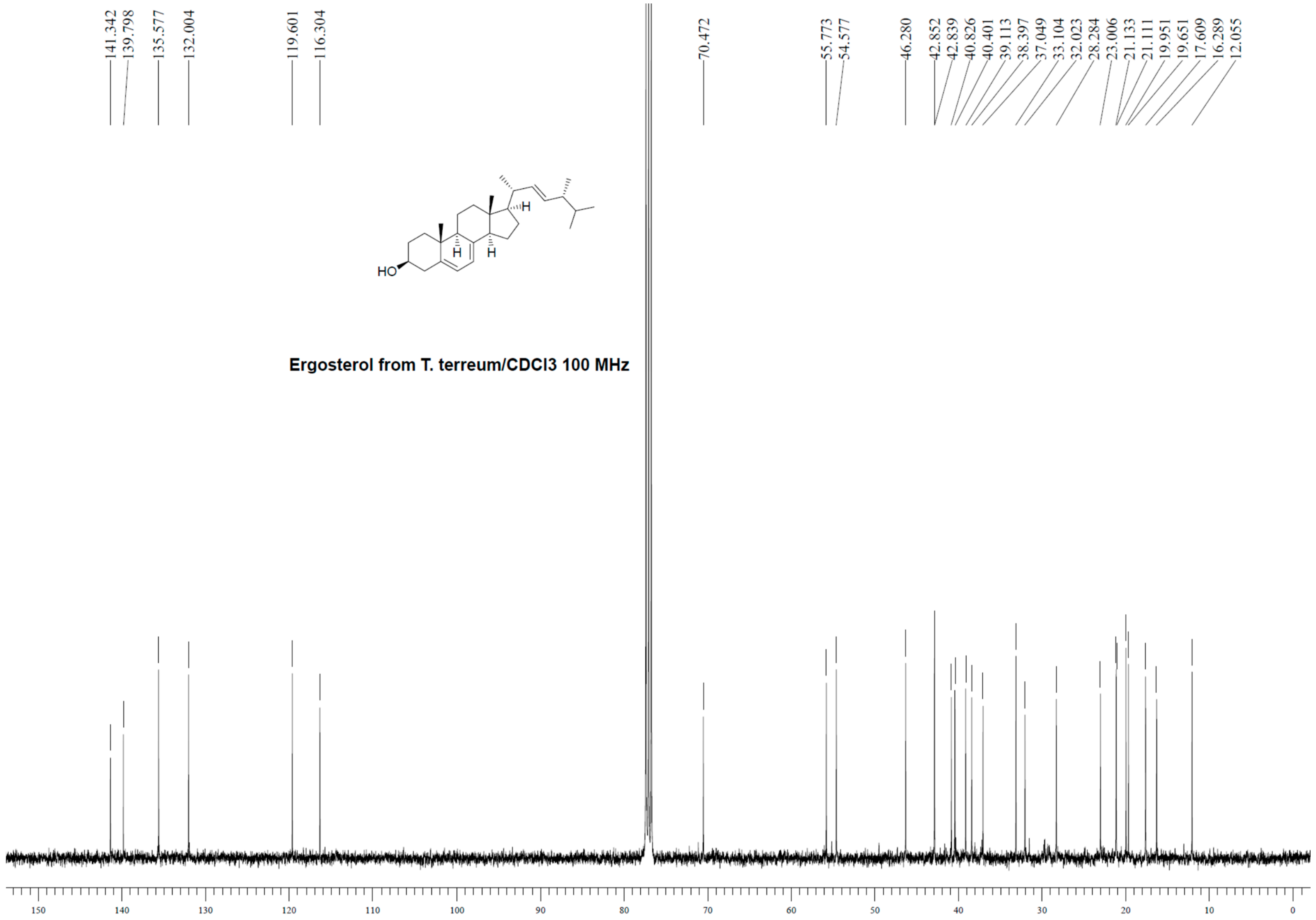

Figure S11: ^{13}C NMR spectrum of ergosterol extracted from *T. terreum* _Bz.

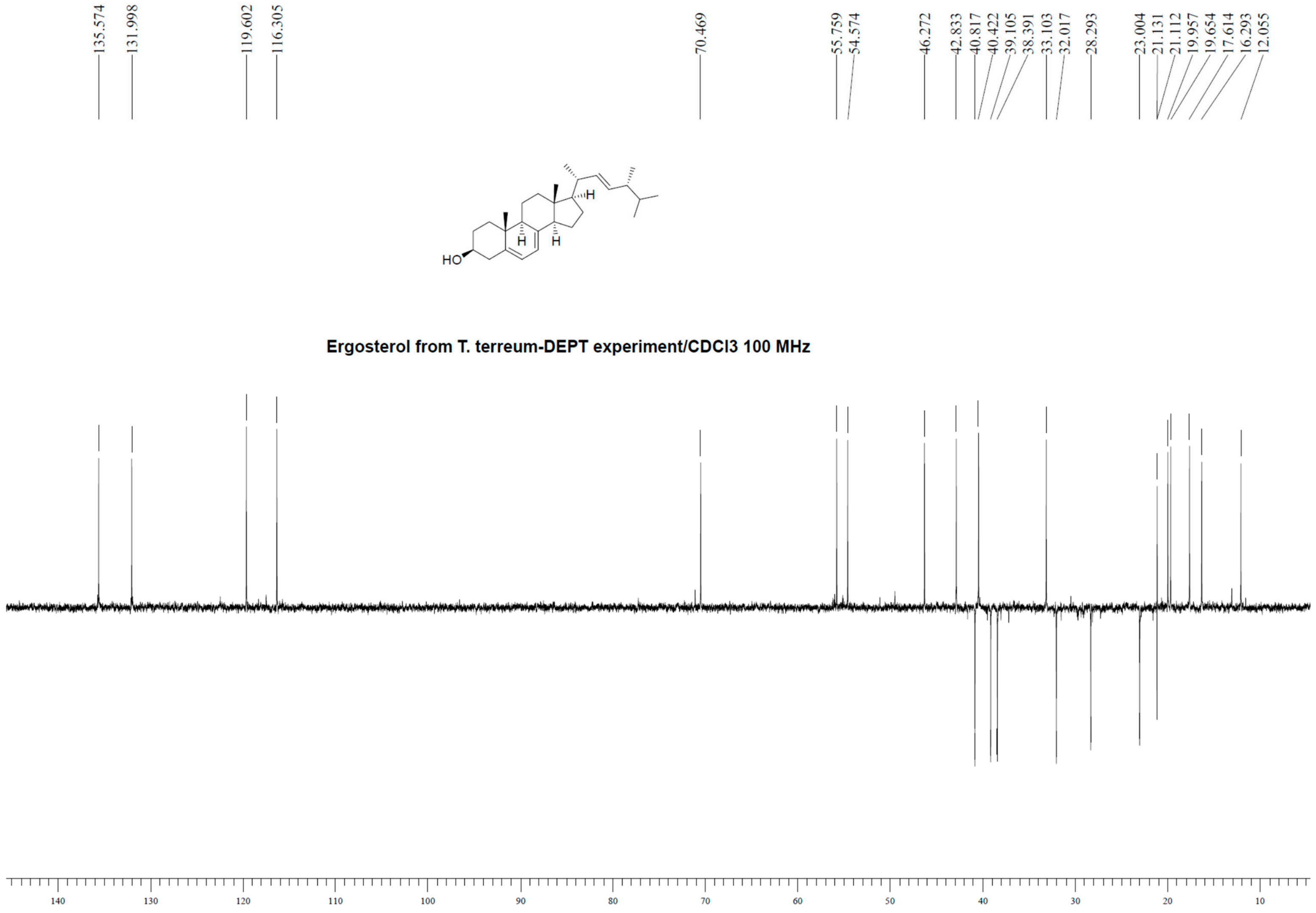

Figure S12: ^{13}C DEPT135 NMR spectrum of ergosterol extracted from *T. terreum* _Bz.

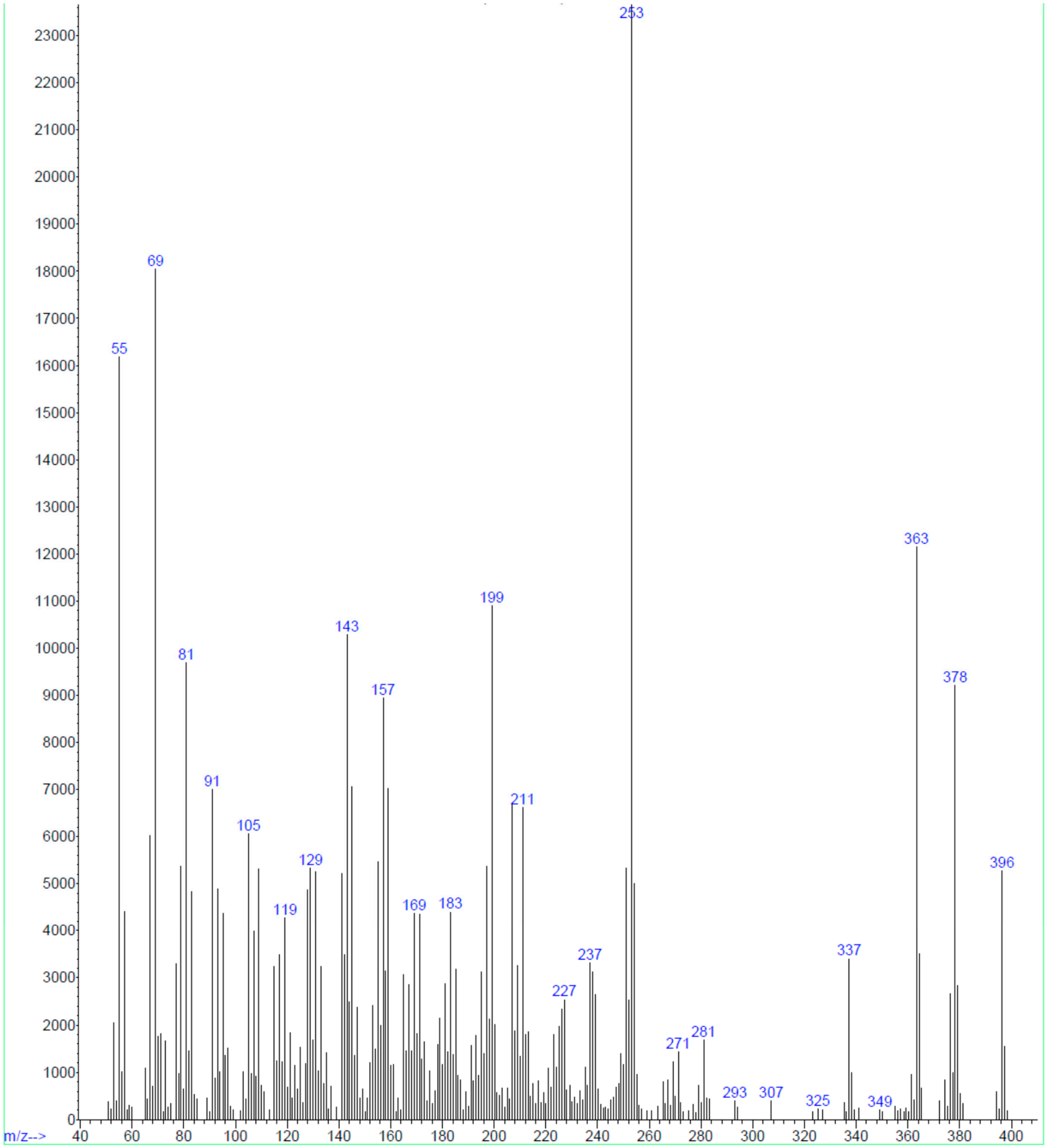

Figure S13: GC-MS spectrum of ergosterol extracted from *T. terreum* _Bz

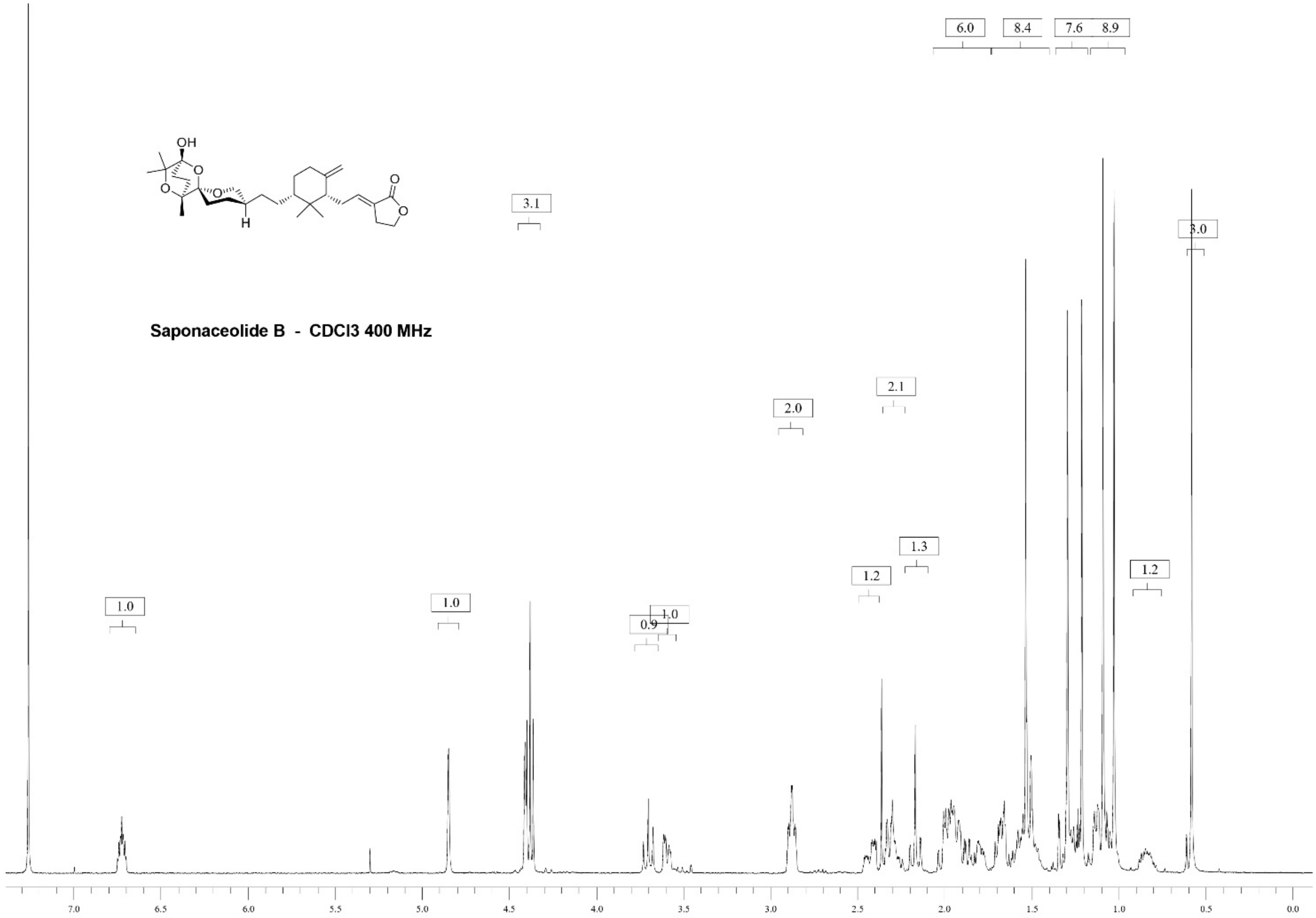

Figure S14: ^{1}H NMR spectrum of the saponaceolide B (**1**) sample obtained in the present investigation.

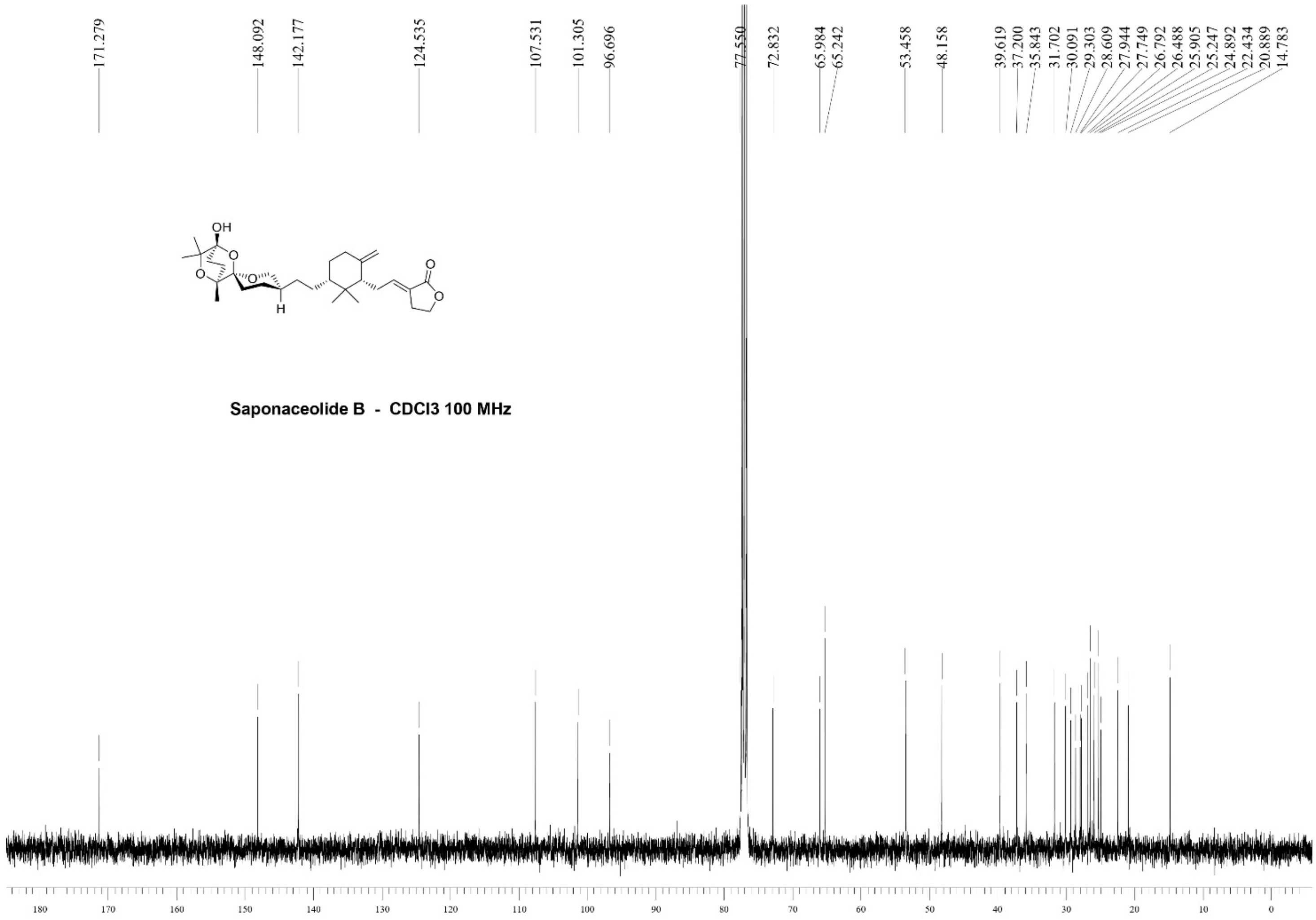

Figure S15: ^{13}C NMR spectrum of the same sample.

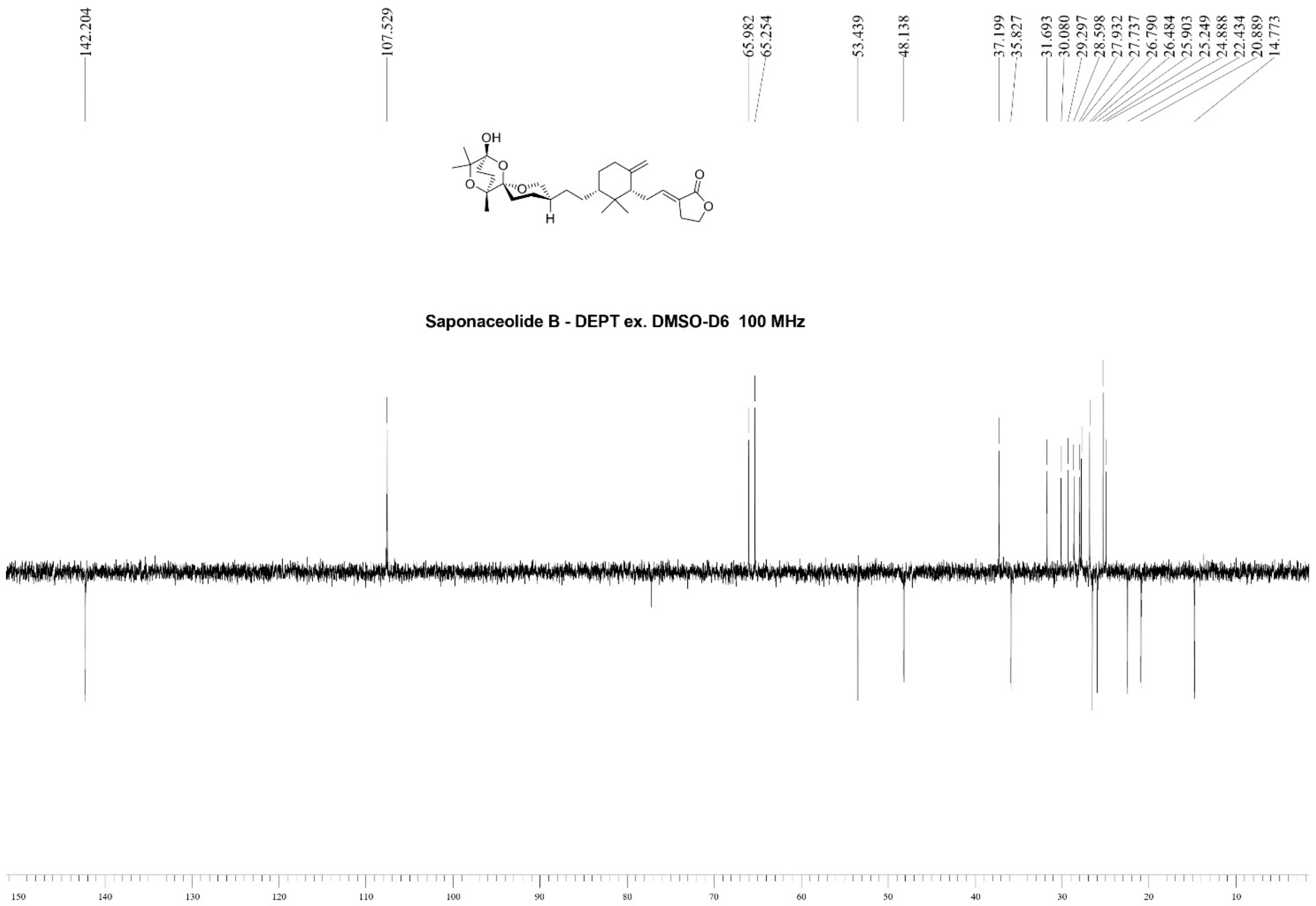

Figure S16: ^{13}C DEPT135 NMR spectrum of the same sample.

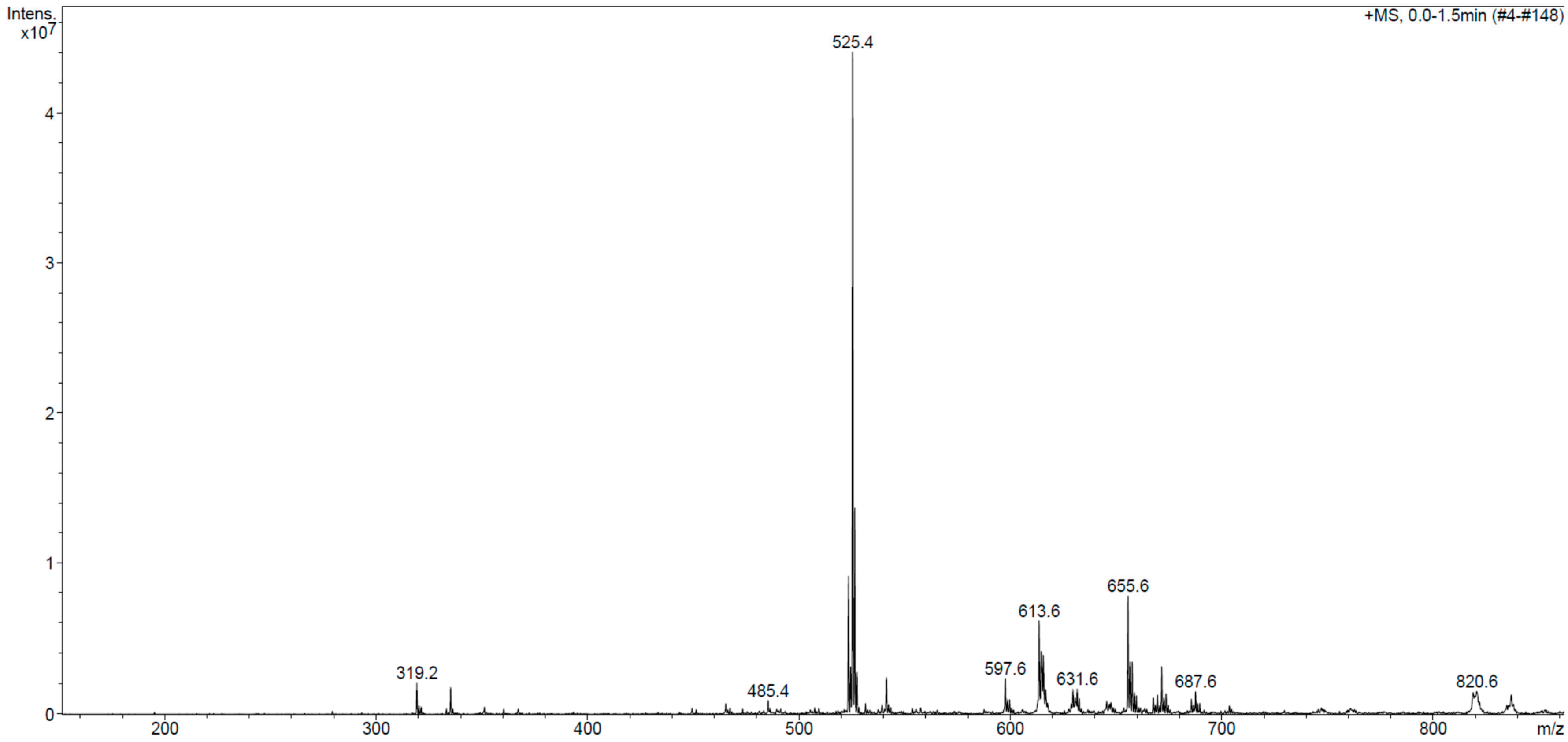

Figure S17: Low resolution ESI^{+} MS spectrum of the same sample.

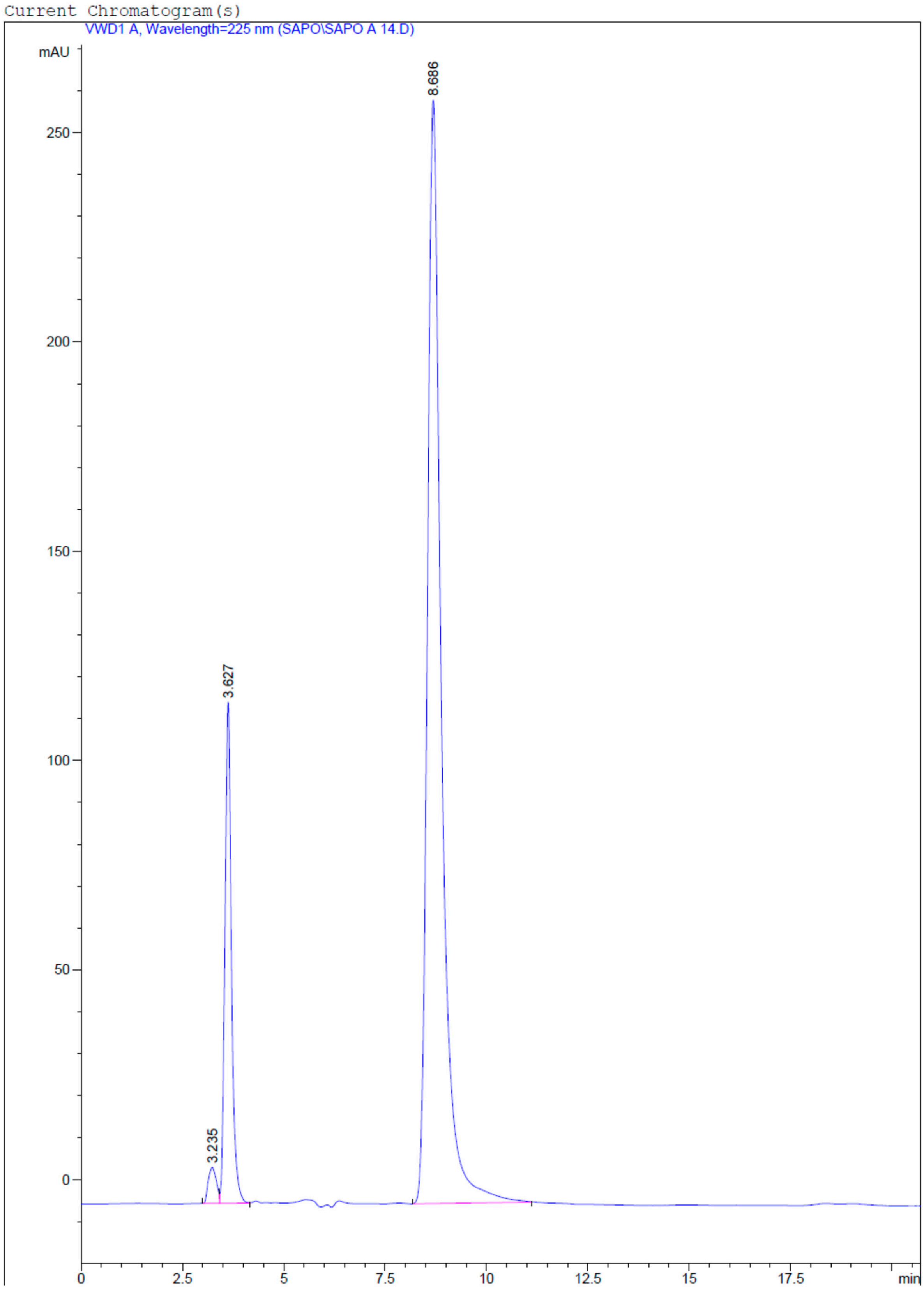

Figure S18: Direct-phase HPLC chromatogram (UV, 225 nm) of a reference standard of saponaceolide A

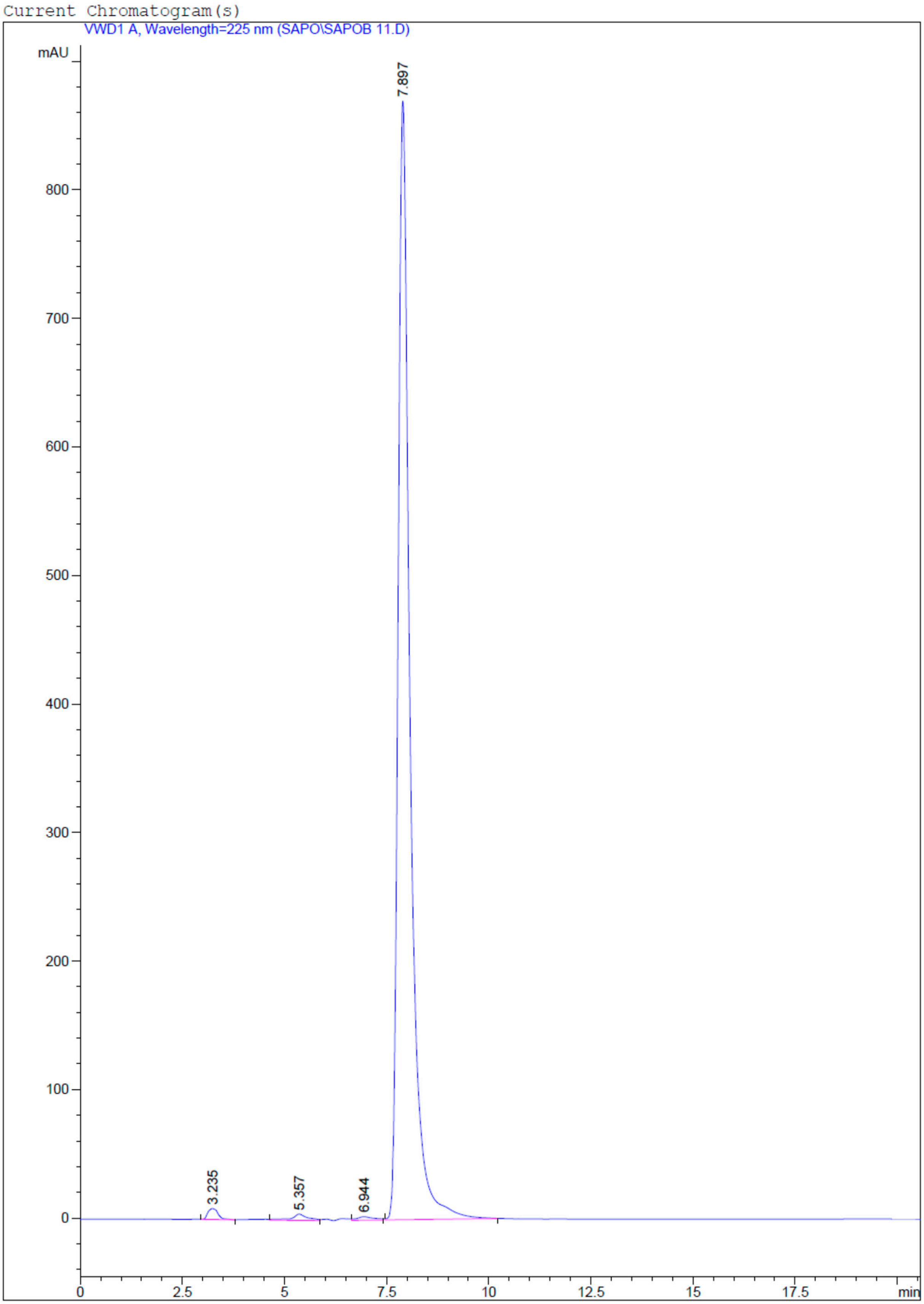

Figure S19: Direct-phase HPLC chromatogram of a reference standard of saponaceolide B.

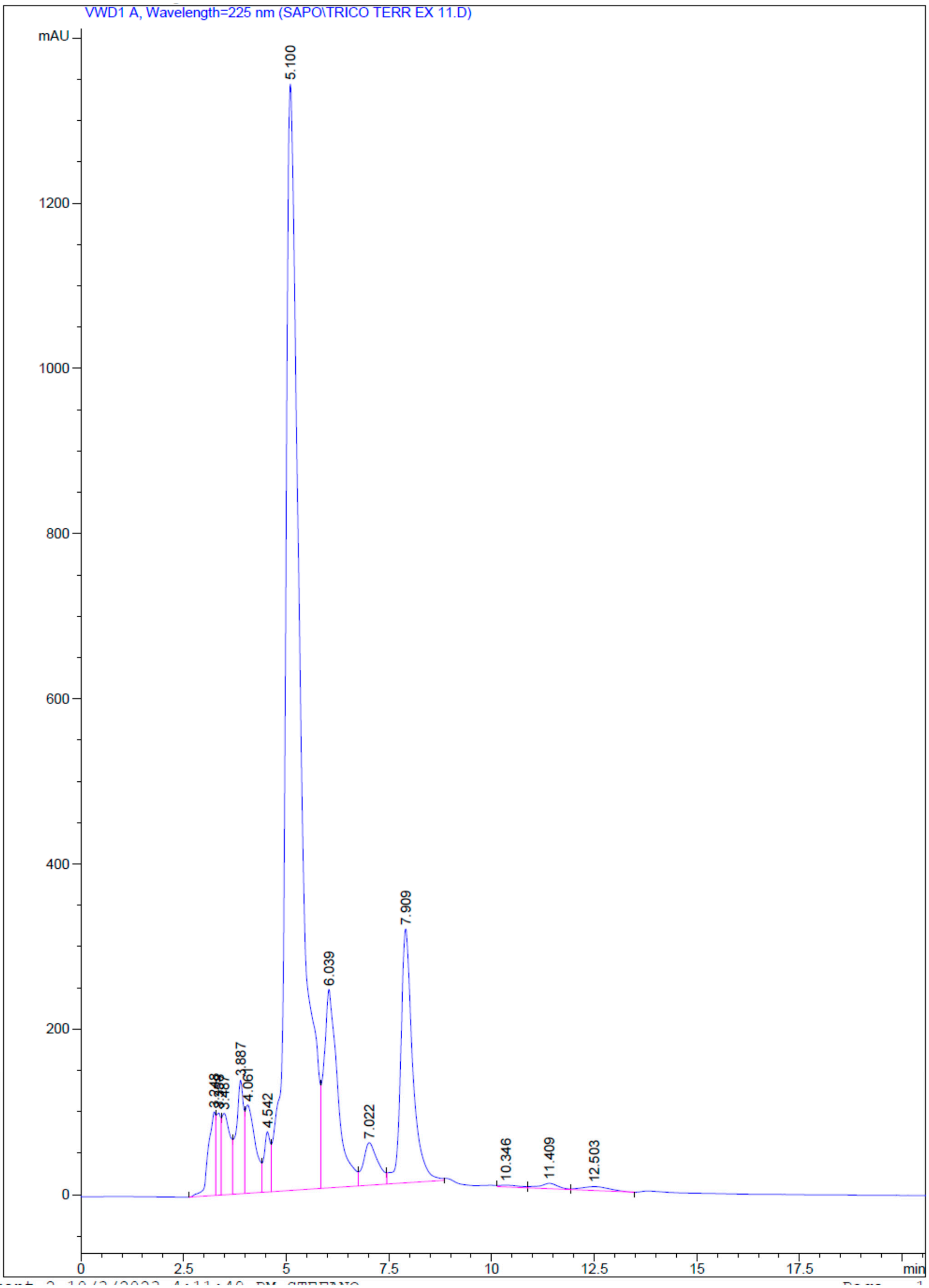

Figure S20: Direct-phase HPLC chromatogram of *T. terreum*_Bz.

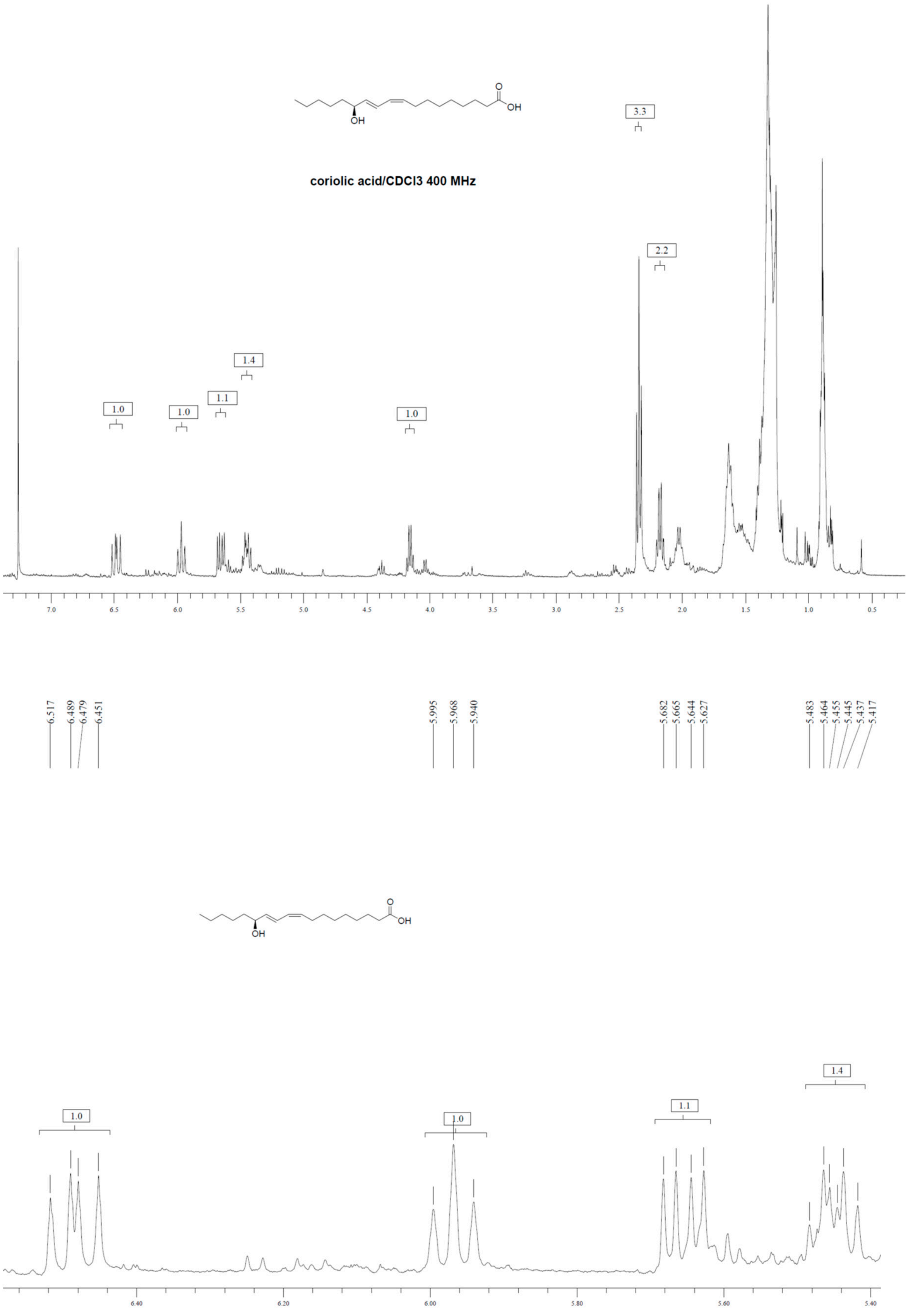

Figure S21: ^{1}H NMR spectrum of the coriolic acid extracted from *T. terreum* _Bz; below the expansion of the vinylic protons signals

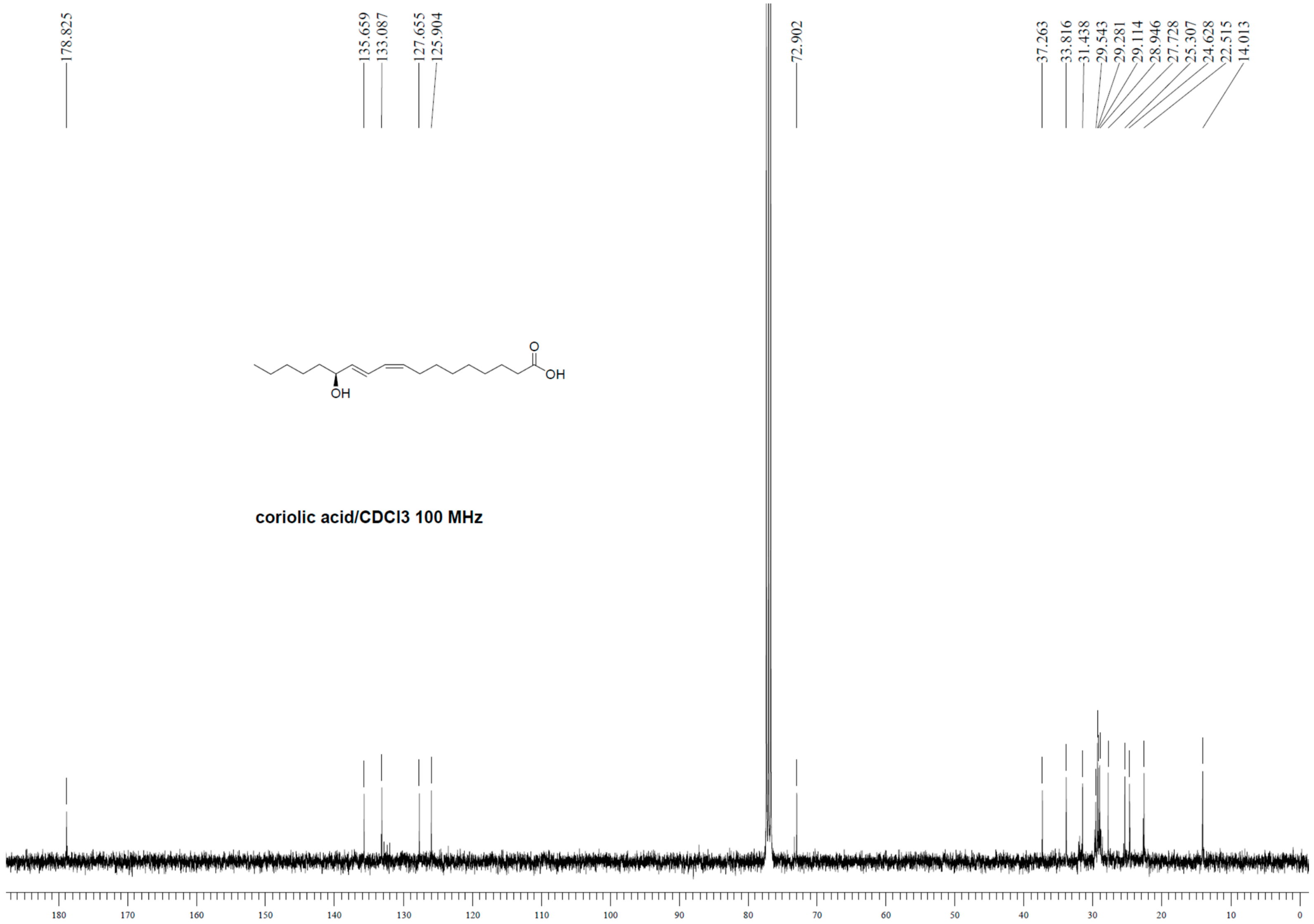

Figure S22: ^{13}C NMR spectrum of the coriolic acid extracted from *T. terreum* _Bz

Acquisition Parameter

Ion Source Type	ESI	Mass Range Mode	Std/Normal	Ion Polarity	Positive	Alternating Ion Polarity	off
Scan Begin	50 m/z	Scan End	950 m/z	Averages	5 Spectra	Accumulation Time	32 µs
Capillary Exit	128.5 Volt	Skim 1	40.0 Volt	Trap Drive	55.0	Auto MS/MS	off

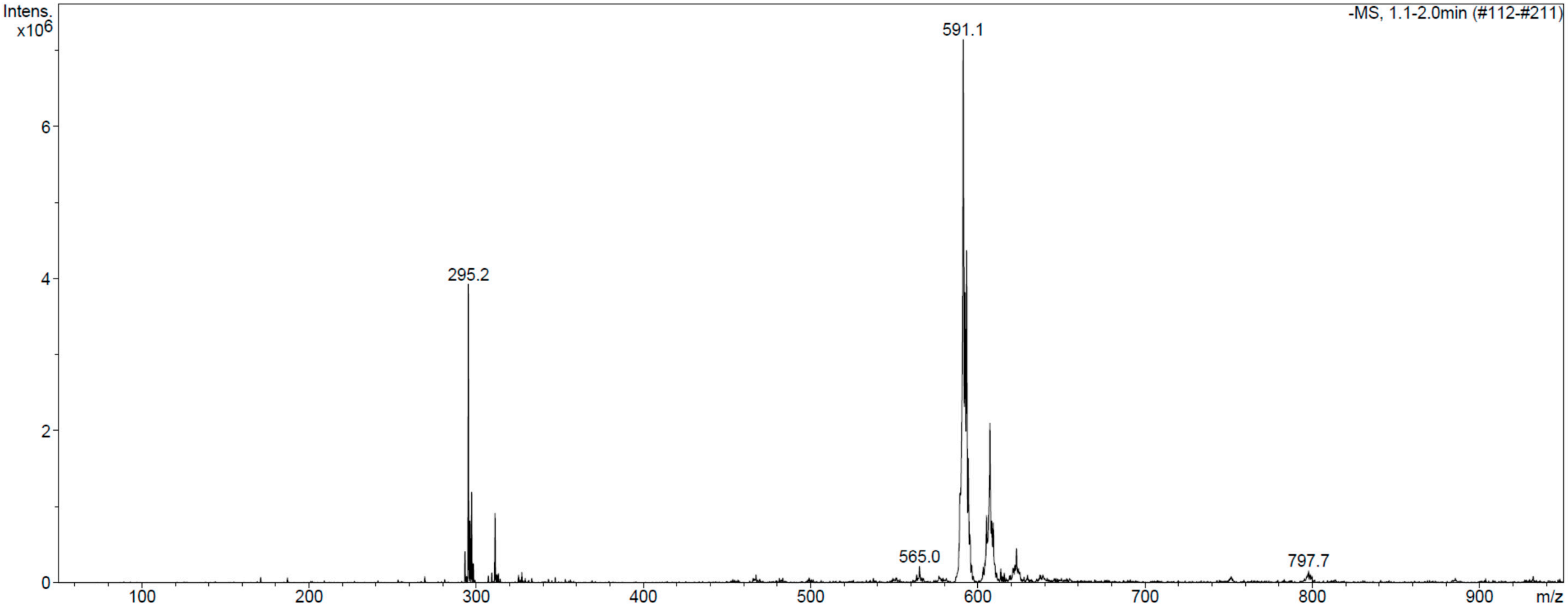

Bruker Daltonics DataAnalysis 3.1 printed: 01/31/24 17:08:39 Page 1 of 1

Figure S23: ESI-MS spectrum of the coriolic acid extracted from *T. terreum* _Bz
